# Hyperbaric oxygen augments susceptibility to *C. difficile* infection by impairing gut microbiota ability to stimulate the HIF-1α-IL-22 axis in ILC3

**DOI:** 10.1080/19490976.2023.2297872

**Published:** 2024-01-02

**Authors:** José L. Fachi, Laís. P. Pral, Helder C. Assis, Sarah Oliveira, Vinícius R. Rodovalho, Jefferson A. C. dos Santos, Mariane F. Fernandes, Valquíria A. Matheus, Renata Sesti-Costa, Paulo J. Basso, Marina Flóro e Silva, Niels O. S. Câmara, Selma Giorgio, Marco Colonna, Marco A. R. Vinolo

**Affiliations:** a Department of Pathology and Immunology, Washington University School of Medicine, St. Louis, MO, USA; b Department of Genetics and Evolution, Microbiology and Immunology, Institute of Biology, University of Campinas, Campinas, Brazil; c Hematology and Hemotherapy Center, University of Campinas, Campinas, Brazil; d Department of Immunology, Institute of Biomedical Sciences, University of São Paulo, São Paulo, Brazil; e Department of Animal Biology, Institute of Biology, University of Campinas, Campinas, Brazil; f Experimental Medicine Research Cluster, Institute of Biology, University of Campinas, Campinas, Brazil; g Obesity and Comorbidities Research Center (OCRC), University of Campinas, Campinas, Brazil

**Keywords:** Hyperbaric oxygen, microbiota, butyrate, clostridioides difficile, innate lymphoid cells, ILC3, HIF-1

## Abstract

Hyperbaric oxygen (HBO) therapy is a well-established method for improving tissue oxygenation and is typically used for the treatment of various inflammatory conditions, including infectious diseases. However, its effect on the intestinal mucosa, a microenvironment known to be physiologically hypoxic, remains unclear. Here, we demonstrated that daily treatment with hyperbaric oxygen affects gut microbiome composition, worsening antibiotic-induced dysbiosis. Accordingly, HBO-treated mice were more susceptible to *Clostridioides difficile* infection (CDI), an enteric pathogen highly associated with antibiotic-induced colitis. These observations were closely linked with a decline in the level of microbiota-derived short-chain fatty acids (SCFAs). Butyrate, a SCFA produced primarily by anaerobic microbial species, mitigated HBO-induced susceptibility to CDI and increased epithelial barrier integrity by improving group 3 innate lymphoid cell (ILC3) responses. Mice displaying tissue-specific deletion of HIF-1 in RORγt-positive cells exhibited no protective effect of butyrate during CDI. In contrast, the reinforcement of HIF-1 signaling in RORγt-positive cells through the conditional deletion of VHL mitigated disease outcome, even after HBO therapy. Taken together, we conclude that HBO induces intestinal dysbiosis and impairs the production of SCFAs affecting the HIF-1α-IL-22 axis in ILC3 and worsening the response of mice to subsequent *C. difficile* infection.

## Introduction

Hyperbaric oxygen therapy (HBO) is a well-established technique that involves exposure to pure oxygen in a special high-pressure chamber, and has been used to promote wound healing, ameliorate post-ischemic and post-inflammatory injuries, and treat bacterial and fungal infections.^1–3^ Increased tissue oxygen perfusion, as achieved by HBO, improves fibroblast growth, angiogenesis, and phagocytic capacity of leukocytes during tissue inflammation.^1,4^ Whether HBO can also be beneficial for intestinal homeostasis and response to infectious/inflammatory stimuli is not clear.

During homeostasis, the mammalian intestinal tract, particularly the large intestine, undergoes physiological hypoxia.^5^ Low oxygen tension is not confined to the intestinal lumen; it is also present in the epithelial layer and the lamina propria (LP).^6^ This hypoxic environment regulates the metabolism of intestinal epithelial cells, enhances barrier integrity, and modulates immune responses in the LP.^6,7^ In addition, intestinal hypoxia has a direct influence on microbiota composition and functions.^8^ The two dominant phyla of bacteria in the lumen, *Firmicutes* and *Bacteroidetes*, representing 90% of the human gut microbiome, are primarily obligate anaerobes.^9^ The reduction of these microbes, coupled with an increase in subdominant facultative anaerobes or the emergence of uncommon aerobes, disrupts microbiome diversity and leads to dysbiosis.^10,11^ Consistently, lower levels of *Firmicutes* and *Bacteroidetes* have been observed in patients with inflammatory bowel disease (IBD).^12–14^ Similarly, it has been reported that increasing tissue oxygenation alters the composition of the gut microbiota in mice^15^ and patients on mechanical ventilation.^16^ This effect has been correlated with an increased mortality rate.^15–19^ However, some studies have suggested that HBO may be beneficial for the treatment of IBD due to its anti-inflammatory effects.^20–22^

Here, we found that HBO exposure accentuates dysbiosis and impairs mucosal immunity, thereby increasing host susceptibility to *Clostridioides difficile* infection (CDI), the most common antibiotic-associated colitis. These observations are closely linked to a decline in the levels of microbiota-derived short-chain fatty acids (SCFA), notably butyrate. Oral supplementation with butyrate reduces intestinal inflammation and improves epithelial barrier function during CDI in HBO-treated mice. However, butyrate failed to attenuate the severity of CDI in mice lacking HIF-1α in RORγt-positive cells. In contrast, the conditional overexpression of HIF-1α within these cells mitigated CDI in mice, whether they were infected or additionally underwent HBO treatment. We validated the role of SCFA butyrate in regulating group 3 innate lymphoid cell (ILC3) responses through HIF-1α signaling in an *in vitro* setting. Finally, these results revealed that HBO impairs host immunity against *C. difficile* infection by decreasing microbiota-derived butyrate production and its role in stimulating the HIF-1α-IL-22 signaling axis in ILC3 (**graphical abstract**).

## Results

### HBO reduces the intestinal length and cytokines production in the gut

**Figure 1.**
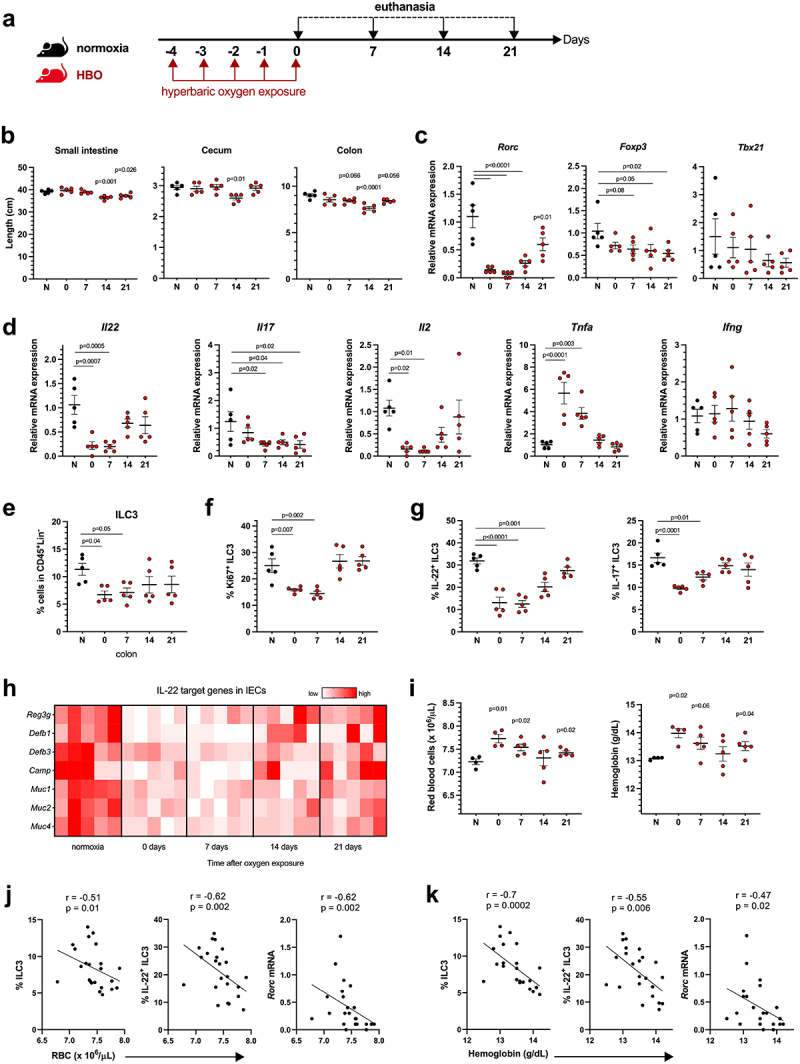
Hyperbaric oxygen modulates intestinal type 3 immunity (a) experimental design illustrating murine treatment with 100% oxygen at 2.5 atmospheres (ATA) for 90 minutes daily over a 5-day period. (b) measurement of intestinal length in mice subjected to hyperbaric oxygen (HBO) treatment. *N* = 5. (c, d) dynamics of relative mRNA expression levels in percoll-purified colonic lamina propria (LP) lymphocytes at various time points post-HBO treatment. *N* = 5. (e, f) flow cytometry analysis depicting the percentage of live lin^−^CD45^int^ CD90.2^+^RORγt+ ILC3 (e) and Ki67+ ILC3 (f) within the colonic LP. *N* = 5. (g) evaluation of the percentage of IL-17- and IL-22-producing ILC3 in the colon of HBO-treated mice. Cells were stimulated *ex vivo* with 10 ng/mL IL-1β and IL-23. *N* = 5. (h) quantitative polymerase chain reaction (qPCR) analysis illustrating the relative mRNA expression of IL-22-target genes in isolated colonic epithelial cells. *N* = 5. (i) hematocytometric analysis revealing the red blood cell number (left) and hemoglobin concentration (right) after HBO treatment. *N* = 5. (j, k) Spearman’s rank correlation coefficient illustrating the relationships between ILC3, IL-22, and rorc mRNA expression in the colon with red blood cell frequency (j) or hemoglobin content (k) in the bloodstream of HBO-treated mice at different time points. All data are representative of at least two independent experiments and are presented as mean ± SEM.

To assess the impact of HBO on intestinal mucosa homeostasis, mice were placed daily for 90 min for 5 days at 100% oxygen in a 2.5 atmospheres (ATA) hyperbaric chamber and euthanized at days 0, 7, 14, and 21 post-treatments (Figure 1a). The lengths of the colon and small intestine were significantly reduced at 14 and 21 days after HBO compared to untreated mice, as well as the cecum size on day 14 (Figure 1b), but there were no changes in body weight (**Fig. S1a**). Colonic LP lymphocytes from HBO-treated mice had reduced levels of *Rorc* mRNA and, to a lesser extent, *Foxp3*, but not *Tbx21* (Tbet), compared to control mice in normoxia (Figure 1c). HBO also reduced the expression of *Il22*, *Il17*, and *Il2* in these cells, while *Tnfa* was increased and *Ifng* was unaffected (Figure 1d).

To obtain a more detailed picture of the effects of HBO on colonic lymphocytes, we separately examined innate lymphoid cells (ILC) and T cells. HBO caused a reduction in the frequency and proliferation of colonic RORγt^+^ ILC3 (days 0 and 7) (Figure 1e-f), but did not alter the proportions of CCR6^+^, NKp46^+^ (NCR^+^), or double-negative subsets (**Fig. S1b)**, as well as the frequencies of ILC1 and ILC2 (**Fig. S1c**). ILC3 are known to maintain epithelial barrier integrity through IL-22 and IL-17^23^. We observed a reduction in both cytokines produced by ILC3 after HBO (Figure 1g). In addition, we noted an increase in the frequency of CD3^+^ T cells on days 7 and 14 post treatment (**Fig. S1d,e**) but no impact on the total CD4^+^ T cell frequency. The frequency of RORγt^+^ Th17 cells was relatively higher in HBO-treated mice than in the controls (**Fig. S1e**), whereas the frequencies of CD4^+^ T cells producing IL-17 and IL-22 were reduced (**Fig. S1f**). These mice also exhibited a reduction in Foxp3^+^ T_reg_ on days 0 and 7 (**Fig. S1e**). These data suggest that HBO affects both ILC3 and CD4^+^ T helper cell frequency and function. Intestinal epithelial cells (IECs) from the colon of HBO-treated mice showed a reduction in the expression of mucins and antimicrobial peptide genes compared with IECs from control mice (Figure 1h), thus indicating a deficiency in IL-22 signaling^24,25^ and corroborating previous evidence regarding the impact of HBO on the intestinal mucosa.

To validate the impact of HBO treatment on the gut through increased tissue oxygen diffusion, we also analyzed blood cells using an automatic hemocytometer counter at various time points after HBO sessions. HBO increased the red blood cell numbers as well as the total amount of hemoglobin shortly after treatment (Figure 1i, **S1g**), indirectly indicating increased oxygen transport in the bloodstream.^26^ The blood platelet and leukocyte counts were unaffected (**data not shown**). Spearman’s rank correlation coefficient showed that red blood cell numbers and hemoglobin concentrations were negatively correlated with ILC3 frequency, IL-22 production, and *Rorc* mRNA expression (Figure 1j, k). Overall, these data indicate that hyperbaric oxygenation reduces the abundance and function of IL-22-producing ILC3 and, to some extent, diminishes CD4+ T cell responses.

### HBO alters the composition of the intestinal microbiota

**Figure 2.**
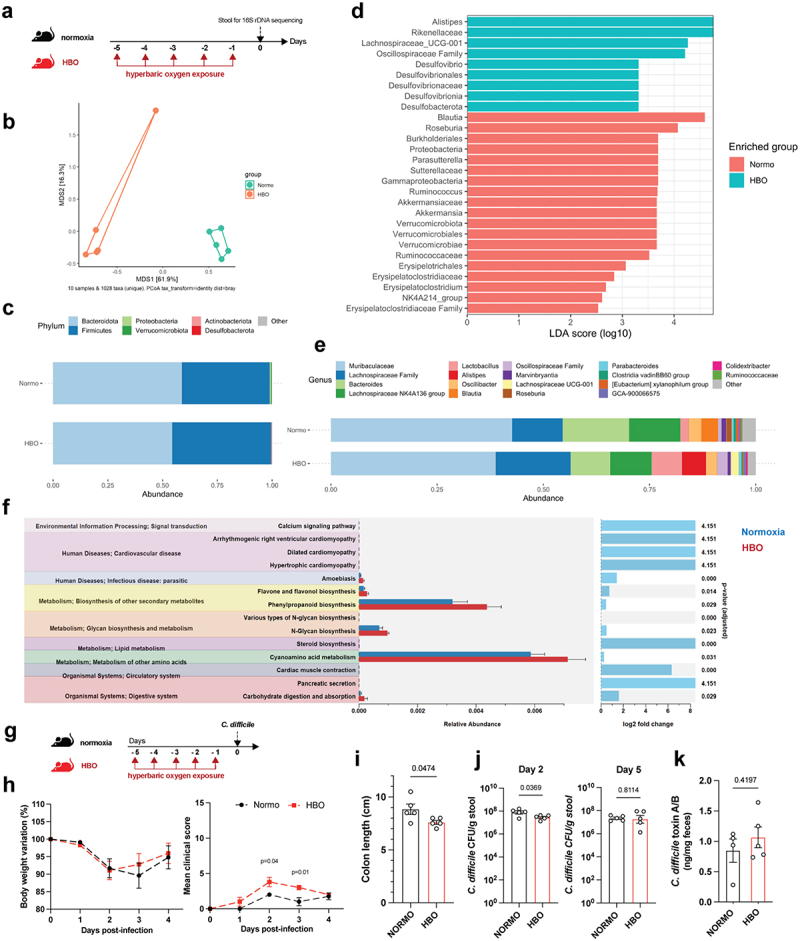
Hyperbaric oxygen modulates gut microbiota composition in mice. (a) experimental framework illustrating the hyperbaric oxygen (HBO) treatment regimen for subsequent analysis of the intestinal microbiome. Mice were subjected to hyperbaric 100% oxygen daily for 90 minutes over a 5-day period. (b) Principal coordinate analysis (PCoA) plot depicting Bray-Curtis dissimilarity index, distinguishing normoxic (green) and HBO-treated (orange) mice. *N* = 5. (c) taxonomy bar plots at the phylum level for normoxic and HBO-treated mice. *N* = 5. Individual plots can be referenced in fig. S2d. (d) linear discriminant analysis effect size (LEfSe) results, visually represented as an effect size (LDA score) bar plot, aiding in the identification of discriminative taxa between experimental groups. *N* = 5. (e) taxonomic bar plots at the genus level. *N* = 5. Individual plots are available in fig. S2e. (f) phylogenetic investigation of communities by reconstruction of unobserved states (PICRUSt2) analysis, predicting functional abundances based on marker gene sequences. *N* = 5. (g) experimental scheme detailing HBO treatment and subsequent *C. difficile* infection (CDI). *N* = 5. (h) evaluation of body weight variation (left) and clinical scores (right) for normoxic and HBO-treated mice post-CDI. *N* = 5. (i-k) assessment of colon length on day 7 post-infection (i), *C. difficile* colony-forming unit (CFU) counts on days 2 (left) and 5 (right) post-infection (j), and quantification of *C. difficile* toxins A and B in luminal contents on day 5 post-infection (k). *N* = 5. All data are presented as the mean ± SEM.

The intestinal lumen constitutively exhibits low levels of oxygen that maintain commensal microbiota, which conversely sustains intestinal hypoxia.^5,27^ Considering the alterations in the mucosal immune cells of mice treated with HBO, we analyzed the effect of HBO on the intestinal microbiota. For this purpose, we sequenced the V3 and V4 regions of the bacterial 16S rDNA extracted from feces of mice treated with or without hyperbaric oxygen for five consecutive days (Figure 2a). According to β-diversity, we observed significant differences between groups, as shown by the Bray-Curtis dissimilarity ratio (Figure 2b) and Weighted and Unweighted UniFrac distances (**Fig. S2a,b**), indicating a distinct gut microbiome composition. No significant difference in α-diversity was detected between the microbiota of normoxic and HBO-treated mice (**Fig. S2c**). Using ALDEX2, a method of differential abundance analysis, we found a significant reduction in *Verrucomicrobiota* and *Proteobacteria* and enrichment of *Desulfobacterota* at the phylum level in HBO-treated mice (Figure 2c ; Fig. S2d). At the genus level, we found an increased abundance of *Alistipes*, *Lachnospiraceae* (*UCG-001*), *Desulfovibrio* and *Erysipelatoclostridium* in the HBO group, while a reduction was observed for *Akkermansia*, *Parasutterella*, *Ruminococcus*, *Roseburia* and *Blautia* (Figure 2d, e; Fig. S2e-g). *Akkermansia* is a genus of strict anaerobic bacteria that colonizes the gastrointestinal tract and regulates the mucus layer and inflammatory/immune processes.^28^ *Ruminococcus* and *Roseburia* are genera of anaerobic bacteria important for metabolism of complex polysaccharides.^29^ On the other hand, *Alistipes* is a genus of bacteria that colonizes the intestinal mucosa and is also known to be resistant to a variety of antibiotics.^30^ *Alistipes* is elevated in patients with anxiety and depression,^31^ and in mice exposed to a high-fat diet.^32^ Taken together, these differences between the microbiota of normoxic and HBO-treated mice imply a potential influence of HBO on metabolism. This aspect is underscored by the results of the PICRUSt2 analysis (Phylogenetic Investigation of Communities by Reconstruction of Unobserved States) (Figure 2f), which predicted changes in metabolism and carbohydrate digestion and uptake associated with HBO induced microbiota.

### HBO-exposed mice are highly susceptible to *C. difficile* infection

To assess whether these HBO-dependent changes in the gut affect host susceptibility to enteric infections, we tested the *Clostridioides difficile* infection (CDI) model, which is known to be a consequence of microbial dysbiosis.^33–35^ Mice were treated with hyperbaric oxygen for 5 days and then infected one day later (Figure 2g). Using this approach, mice developed only mild symptoms of the disease. Interestingly, HBO-treated mice showed higher clinical scores and colon shortening (Figure 2h, i), indicating an increased susceptibility to CDI. In addition, despite lower *C. difficile* counts on day 2 post-infection (p.i.) in the HBO group, we did not observe differences in the number of colony forming units (CFU) on day 5 p.i., as well as in *C. difficile* toxin A/B levels (Figure 2j, k). This suggested that oxygen-induced dysbiosis is sufficient to promote CDI without affecting *C. difficile* colonization or toxin production.

**Figure 3.**
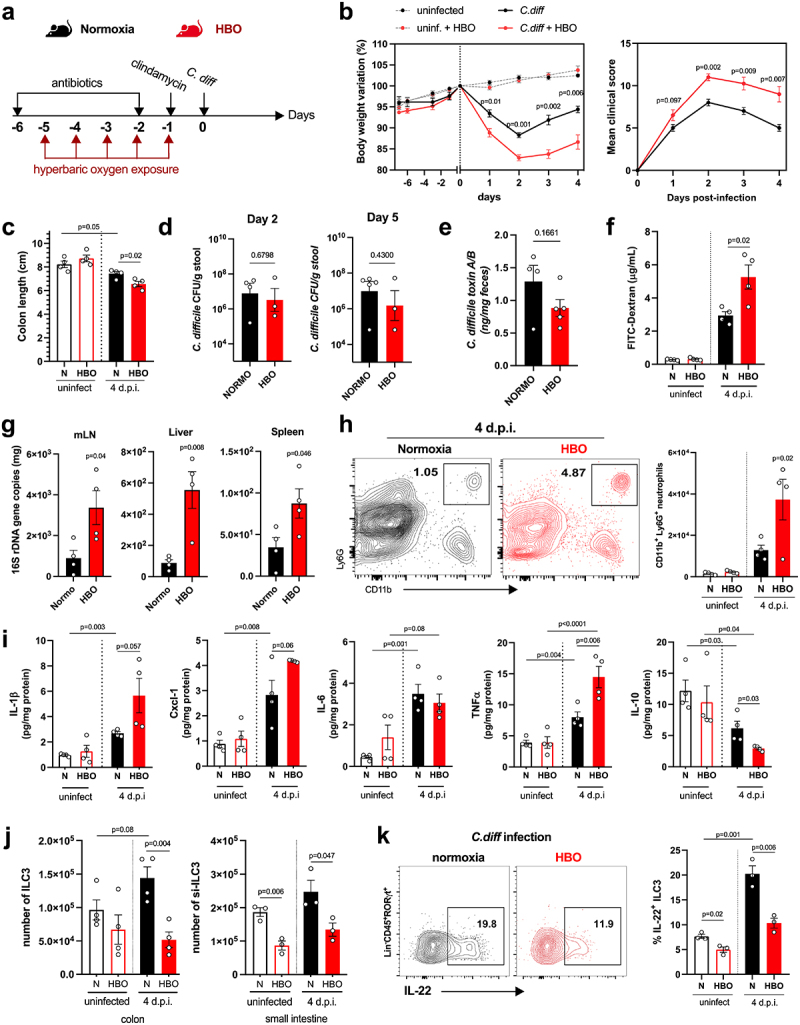
Increased susceptibility to *C. difficile* infection in HBO-exposed mice. (a) experimental design detailing the *C. difficile* infection (CDI) model with hyperbaric oxygen (HBO) treatment. Mice were subjected to a combination of antibiotics in drinking water for 4 days, followed by a single intraperitoneal dose of clindamycin. Subsequently, they were either infected or not with 10^8^ CFU of *C. difficile* strain VPI 10,463. HBO sessions were conducted daily for 90 minutes during the 5 days preceding the infection (days −5 to − 1). (b) body weight variation (left) and clinical scores (right) recorded during CDI. *N* = 8, with antibiotics administered to uninfected groups, although they were not subjected to a *C. difficile* challenge. (c) assessment of colon length on day 4 post-infection. *N* = 4. (d) quantification of *C. difficile* colony-forming units (CFU) in the feces of normoxic or HBO-treated mice on days 2 (left) and 5 (right) post-infection. *N* = 3–5. (e) quantification of *C. difficile* toxin a and B luminal contents on day 5 post-infection. *N* = 5. (f) analysis of intestinal epithelial permeability through serum quantification of FITC-dextran after 4 hours of gavage. *N* = 4. (g) relative bacterial 16S rDNA quantification in mesenteric lymph nodes (mLN), liver, and spleen by qPCR on day 4 post-infection. *N* = 4. (h) absolute numbers of live CD45^+^CD11b^+^Ly6G^+^ neutrophils in the colonic lamina propria (LP) of uninfected and day 4 infected mice. *N* = 4. (i) quantification of inflammatory cytokines in the proximal colon by enzyme-linked immunosorbent assay (ELISA). *N* = 4. (j) absolute number of live lin- CD45^int^CD90.2^+^RORγt^+^ ILC3 in the colonic (left) and small intestine (right) LP of uninfected and 4 days post-infection mice. *N* = 3–4. (k) frequency of IL-22-producing ILC3 from normoxic or HBO-treated mice after *ex vivo* stimulation with IL-1β/IL-23 for 3 hours. *N* = 3. All results are representative of at least two independent experiments and are presented as the mean ± SEM.

Next, we treated mice with a cocktail of antibiotics for 4 days, in parallel with HBO, and then either infected or did not infected them with 10^8^ CFU of *C. difficile* strain VPI 10,463 (Figure 3a). All infected mice developed clear signs of disease, and HBO aggravated body weight loss and clinical score (Figure 3b), as well as colon shortening (Figure 3c). No difference in *C. difficile* CFU or toxin A/B concentrations was observed between the groups on days 2 and 5 post-infection (Figure 3d, e). As the VPI 10,463 strain produces high levels of toxin,^36^ we also tested a more clinically relevant strain to confirm the impact of HBO on host susceptibility to CDI. The PCR ribotype 078 strain (RT078) was also capable of inducing signs of disease in mice (**Fig. S3a-c**), despite being milder and delayed compared with VPI 10,463 (Figure 3b). Even in RT078-infected mice, we continued to observe a deterioration in response after HBO treatment (**Fig. S3b-d**). We observed no differences in CFU counts on days 2 and 5 post-infection (**Fig. S3e**), indicating that HBO had no major impact on intestinal colonization by *C. difficile*.

In addition, we noted that HBO impaired the intestinal epithelium permeability to FITC-dextran in VPI 10,463-infected mice (Figure 3f) and increased bacterial translocation, as shown by the quantification of bacterial 16S rDNA levels in the mesenteric lymph nodes (mLN), liver, and spleen (Figure 3g). Similarly, in antibiotic-pre-treated and infected mice, HBO increased the infiltration of CD11b^+^Ly6G^+^ neutrophils in colonic LP (Figure 3h), but not of CD11b^+^F4/80^+^ macrophages and CD11c^+^CD11b^+^ dendritic cells (**Fig. S4a**). HBO was also associated with increased production of pro-inflammatory cytokines such as IL-1β, CXCL1, and TNFα, while IL-10 was reduced (Figure 3i ; Fig. S4b). As expected, CDI caused an accumulation of ILC3 (Figure 3i), but not of ILC1 or ILC2 (**Fig. S4c,d**) in the colon and small intestine LP a few days post-infection. However, the frequency of ILC3, particularly the RORγt^+^ NCR^–^ subtype, in the lamina propria of the colon and small intestine, was diminished in mice treated with HBO, both in the infected and uninfected groups (Figure 3j ; Fig. S4e). Similar results were observed for the ILC3s number was observed in the mesenteric lymph nodes (**Fig. S4f**). HBO treatment also reduced ILC3 production of IL-22 (Figure 3k), IL-17, and IFN-γ (**Fig. S4g,h**) after *ex vivo* stimulation with IL-1β and IL-23, indicating that hyperbaric oxygenation impairs ILC3 responsiveness to stimuli. Controversially, HBO had no effect on the frequency of CD4^+^ and RORγt^+^ T cells in the LP and mesenteric lymph nodes of the small intestine, despite a slight increase in splenic Th17 (**Fig. S5a-c**), which suggests that HBO mostly affects intestinal ILC3 during *C. difficile* infection.

### HBO aggravates antibiotic-mediated dysbiosis and impaired SCFA production

**Figure 4.**
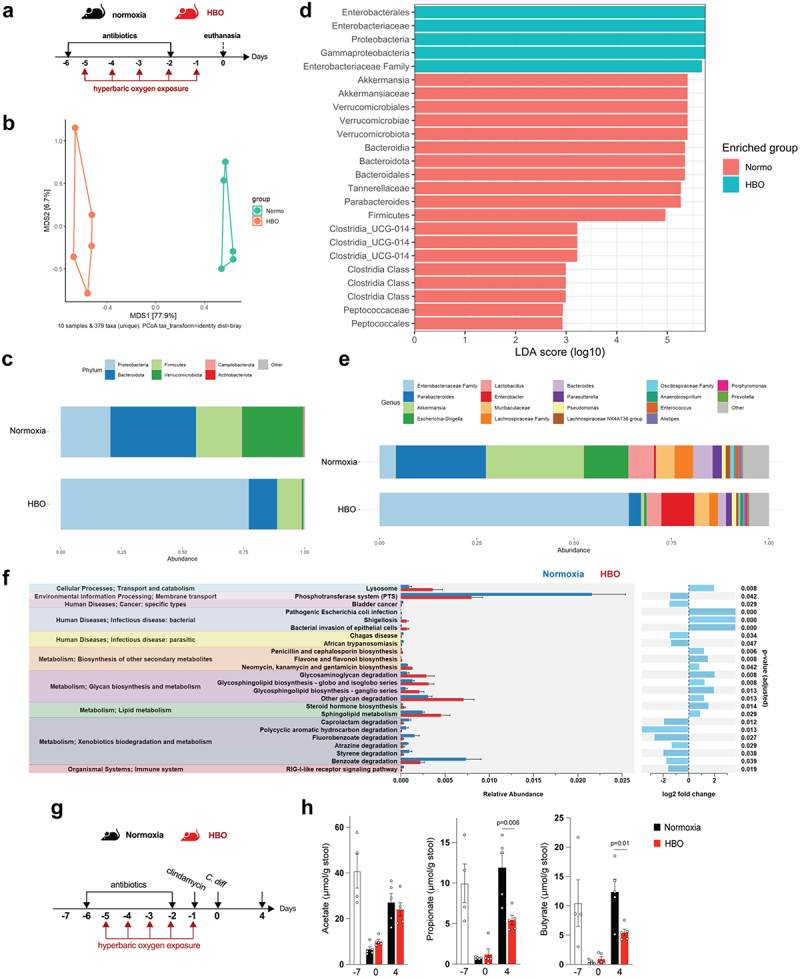
HBO potentiates antibiotic-associated dysbiosis and impairs intestinal SCFA levels. (a) experimental outline detailing the treatment of mice with oral antibiotics and concurrent hyperbaric oxygen (HBO) therapy before fecal 16S rDNA sequencing. (b) microbiome β-diversity analysis based on the Bray-Curtis dissimilarity ratio, represented by a principal coordinate analysis (PCoA) plot. *N* = 5. (c) bar plots depicting 16S rDNA gene reads assigned to taxonomy at the bacterial phylum level in fecal samples from normoxic or HBO-treated mice following antibiotic treatment. *N* = 5, with individual plots available in fig. S6d. (d) linear discriminant analysis effect size (LEfSe) results, visualized as an effect size (LDA score) bar plot, aiding in the identification of discriminative taxa between experimental groups. *N* = 5. (e) taxonomic bar plots at the genus level for normoxic and HBO-treated mouse fecal samples after antibiotic treatment. *N* = 5. Individual plots are detailed in fig. S6e. (f) prediction of metagenomic functional content using phylogenetic investigation of communities by Reconstruction of Unobserved States (PICRUSt2) based on marker gene sequences. *N* = 5. (g) experimental scheme illustrating antibiotic treatment and subsequent *C. difficile* infection (CDI) after HBO therapy. (h) quantification of luminal short-chain fatty acid (SCFA) levels by gas chromatography-mass spectrometry (GC-MS) in the proximal colon of HBO-infected mice. Sample size *N* = 4–5. GC-MS data are representative of at least two independent experiments and are presented as the mean ± SEM.

Next, we investigated whether HBO could aggravate antibiotic-induced dysbiosis before CDI (Figure 4a). The β-diversity of the microbiota of normoxic and HBO-treated mice was significantly different after antibiotic treatment, as shown by the Bray-Curtis ratio (Figure 4b) and the quantitative weighted and qualitative Unweighted UniFrac distances (**Fig. S6a,b**), whereas there were no significant differences in the α-diversity (**Fig. S6c**). HBO therapy was associated with a significant expansion of *Proteobacteria* with enrichment of components of the *Enterobacteriaceae* family (Figure 4c-e ; Fig. S6d-g). Overgrowth of *Proteobacteria* is one of the most clinically significant imbalances in the gut microbiome and is commonly associated with moderate-to-severe ulcerative colitis and Crohn’s colitis in patients.^37–39^ In contrast, normoxic mice were enriched in components of the phyla *Firmicutes*, *Bacteroidota*, and *Verrucomicrobiota*, particularly in the families *Akkermansiaceae*, *Tannerellaceae and Peptococcaceae* (Figure 4c-e ; Fig. S6d-g). Taken together, these data corroborate that HBO aggravates antibiotic-induced dysbiosis in a specific manner.

We performed PICRUSt2 analysis to predict the functional pathways altered by HBO on dysbiosis. HBO-treated mice showed increased glycan degradation and lipid metabolism, whereas normoxic mice showed improved xenobiotic biodegradation and metabolism (Figure 4f). In addition, because members of *Bacteriodota*, *Firmicutes*, and *Verrucomicrobiota* phyla produce short-chain fatty acids (SCFA) – acetate, propionate, and butyrate – through anaerobic fermentation of dietary fibers,^9,40^ we tested whether HBO enhances the impact of antibiotics on SCFA production before and after CDI (Figure 4g). In both groups, luminal levels of short-chain fatty acids (SCFA) in the proximal colon decreased after 4 days of antibiotic treatment but showed a tendency to recover spontaneously a few days later (Figure 4h)^41^. However, a persistent reduction in butyrate and propionate levels was observed in HBO-treated mice after CDI (day 4) (Figure 4h). These results indicate that HBO amplifies the changes induced by antibiotics in both the composition and metabolic function of the microbiota.

### Butyrate attenuates the impact of HBO during CDI

**Figure 5.**
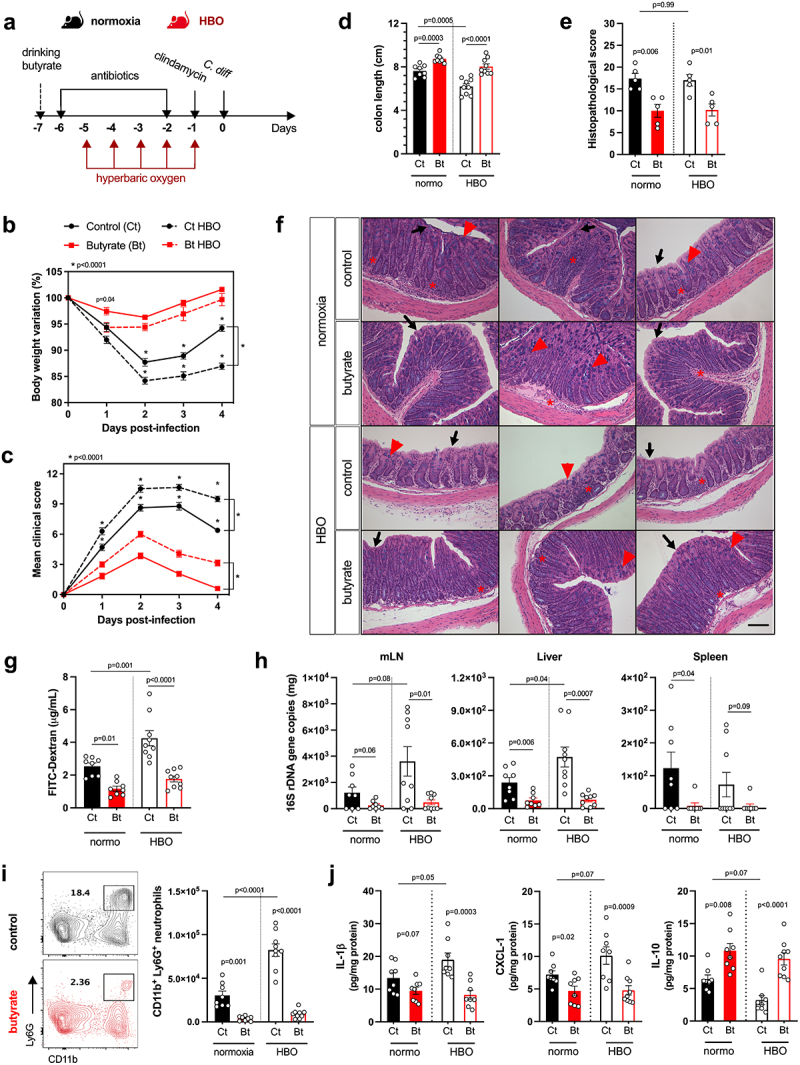
Butyrate supplementation alleviates CDI outcomes in HBO-Treated mice. (a) experimental design illustrating the combination of hyperbaric oxygen (HBO) therapy and oral butyrate treatment in the *C. difficile* infection (CDI) model. (b, c) body weight variation (b) and clinical scores (c) recorded during CDI. *N* = 8–9. (d) colon length on day 4 post-infection. *N* = 8–9. (e, f) quantitative histopathological score (e) and representative H&E-stained images of the colon (f) on day 4 post-infection. Scale bars = 100 µm. *N* = 5. Black arrow indicates epithelial cell necrosis; red arrow indicates goblet cells; red asterisk shows immune cell accumulation. (g) quantification of serum levels of FITC-dextran 4 hours after gavage on day 2 post-infection. *N* = 8–9. (h) relative bacterial 16S rDNA copies detected by qPCR in the mesenteric lymph nodes, liver, and spleen on day 4 post-infection. *N* = 8–9. (i) absolute numbers of live CD45^+^CD11b^+^Ly6G^+^ neutrophils in the colonic lamina propria at 4 days post-infection. *N* = 8. (j) inflammatory cytokine levels in the proximal colon on day 4 post-infection. *N* = 8. All results are derived from at least two independent experiments and are presented as the mean ± SEM.

Butyrate is an energy source for intestinal epithelial cells and contributes to the maintenance of mucosal barrier integrity as well as reduces gut inflammation.^41–45^ Thus, we hypothesized that restoring luminal butyrate levels is sufficient to attenuate HBO-induced susceptibility to CDI. To test this hypothesis, we supplemented the drinking water with butyrate before HBO treatment and infection (Figure 5a). Butyrate reduced body weight loss and improved clinical score (Figure 5b, c) and colon length (Figure 5d), but not small intestine and cecum lengths (**Fig. S7a,b**) in both HBO and normoxic mice. No difference was observed in the content of *C. difficile* toxin A/B in the feces between these groups (**Fig. S7c**), indicating that the protection induced by butyrate was unlikely to be due to an effect on the virulence factors of *C. difficile*. The beneficial effect of butyrate on CDI was confirmed by quantification of histopathological scoring at day 4 p.i. (Figure 5e, f). Epithelial necrosis and immune cell infiltration were reduced, while crypt sizes and number of goblet cells were increased in both HBO and normoxic mice treated with butyrate. We further confirmed that butyrate increased colonic goblet cell and mucopolysaccharide content by periodic acid – Schiff (PAS) staining (**Fig. S7d,e**). Butyrate-mediated improvement of epithelial barrier integrity further extended to intestinal leakage, as measured by FITC-dextran assay (Figure 5g), and translocation of intestinal bacteria to mesenteric lymph nodes (mLN), liver, and spleen (Figure 5h).

Butyrate supplementation reduced infiltration of neutrophils into the colonic LP in both HBO and normoxic mice (Figure 5i), while the abundance of macrophages and dendritic cells remained unchanged (**Fig. S7f**). Decreased inflammatory infiltration was associated with reduced tissue expression of IL-1β, CXCL1, and TNFα, while anti-inflammatory IL-10 was increased and IL-6 remained unchanged (Figure 5j; **S7g**). These data corroborate that HBO-mediated susceptibility to CDI is, at least in part, due to the depletion of butyrate-producing components of the microbiota, although butyrate treatment also attenuated CDI pathology in normoxic conditions.

### Butyrate boosts IL-22-producing ILC3 during CDI

**Figure 6.**
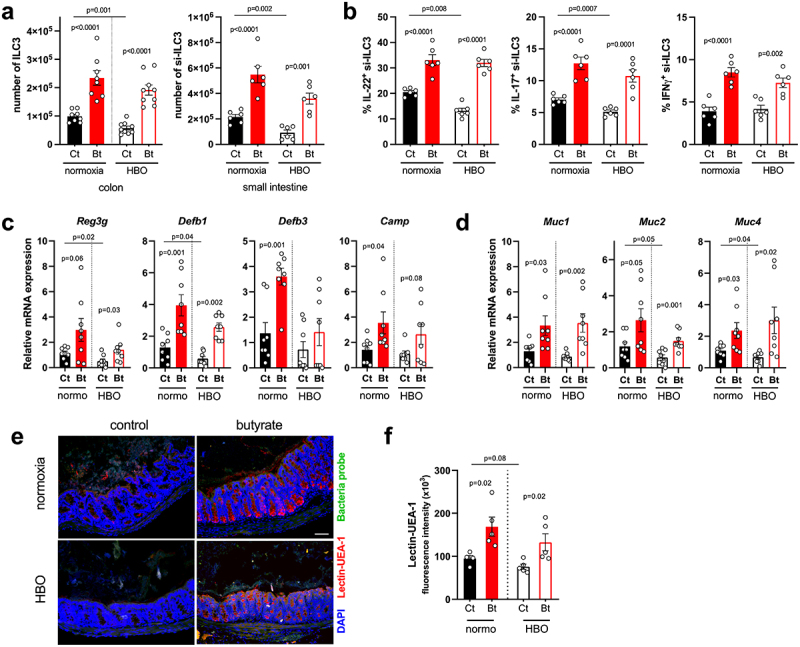
Butyrate enhances ILC3 responses and intestinal IL-22 signaling. (a) absolute numbers of live lin^−^CD45^int^CD90.2^+^RORγt^+^ ILC3 in the colonic (left) and small intestine (right) lamina propria on day 4 post-infection. *N* = 8–9. (b) percentage of IL-22, IL-17, and IFN-γ content in small intestine ILC3 from infected mice after *ex vivo* stimulation with IL-1β/IL-23. *N* = 6. (c, d) relative mRNA expression of antimicrobial peptides (c) and mucin (d) genes in isolated colonic epithelial cells on day 4 post-infection. *N* = 8. (e, f) analysis of mucus density by lectin-UEA-1 *in situ* hybridization in the proximal colon on day 4 post-infection. Blue = DAPI, red = lectin-UEA-1, and green = bacteria probe. Scale bars = 100 µm. *N* = 5. All results are representative of at least two independent experiments and presented as mean ± SEM.

Since HBO affected the expansion and cytokine production of ILC3 in CDI (see Figure 3j, k), we next examined whether butyrate supplementation attenuates this effect. Indeed, butyrate augmented the frequency of ILC3s (mainly CCR6^+^ and NCR^−^ subsets) in the colon and small intestine LP during CDI in both control and HBO-treated mice (Figure 6a; **S7h,i**) as well as their ability to produce IL-22, IL-17, and IFN-γ after *ex vivo* stimulation with IL-1β and IL-23 (Figure 6b). Butyrate had no effect on ILC1, whereas the frequency of ILC2 in the colon and small intestine LP was reduced (**Fig. S7j,k**), which is consistent with a previous report that butyrate suppresses the frequency and function of pulmonary ILC2 in a murine model of asthma.^46^ Since IL-22 stimulates epithelial cell proliferation and production of antimicrobial peptides and mucus,^47^ we hypothesized that the beneficial effect of butyrate on HBO-mediated susceptibility to CDI pathology may be related, in part, to its ability to rescue IL-22-producing ILC3. Accordingly, isolated intestinal epithelial cells of HBO and control mice treated with butyrate showed increased expression of IL-22 target genes encoding antimicrobial peptides (*Reg3g*, *Defb1*, *Defb3* and *Camp*) (Figure 6c) and mucins (*Muc1*, *2* and *4*) (Figure 6d). Fluorescent *in situ* hybridization (FISH) analysis further validated that while HBO-exposed mice had slight mucus depletion in CDI, butyrate restored the mucus layer density (Figure 6e, f). We conclude that butyrate attenuates HBO-induced susceptibility to CDI by restoring ILC3 and IL-22 production, which sustains intestinal epithelial cell defense.

### Butyrate induces ILC3 responses through HIF-1 dependent pathway

**Figure 7.**
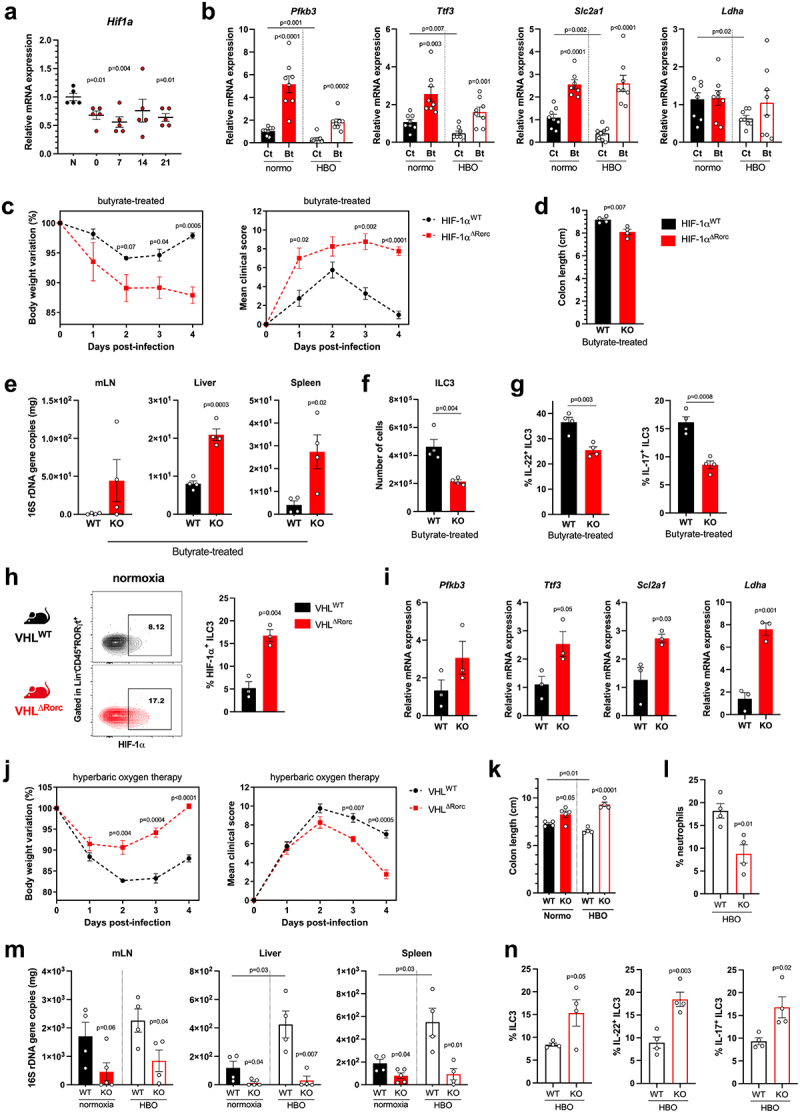
The efficacy of butyrate in conferring protection during CDI relies on the activation of HIF-1α in ILC3. (a) Relative *Hif1a* mRNA expression by percoll-purified lymphocytes from colonic lamina propria at different time points after hyperbaric oxygen (HBO). *N* = 5. (b) HIF-1 target genes mRNA expression by isolated lymphocytes from the colonic lamina propria on day 4 post-infection. *N* = 8. (c) body weight variation (left) and clinical scores (right) of butyrate-treated HIF-1 sufficient (HIF-1α^WT^) or conditional deficient (HIF-1α^ΔRorc^) mice after HBO and CDI. *N* = 4. (d) colon length of butyrate-treated HIF-1α^WT^ and HIF-1α^ΔRorc^ mice after HBO therapy on day 4 post-infection. *N* = 4–5. (e) relative 16S rDNA copies in the mesenteric lymph nodes, liver, and spleen of butyrate-treated HIF-1α^WT^ and HIF-1α^ΔRorc^ mice after HBO on day 4 post-infection. *N* = 8–9. (f, g) absolute number of live lin^−^CD45^int^CD90.2^+^RORγt^+^ ILC3 (f) and its IL-22 and IL-17 content (g) in the colonic lamina propria 4 days post-infection of butyrate-treated HIF-1α^WT^ and HIF-1α^ΔRorc^ mice after HBO. *N* = 4–5. (h, i) HIF-1α intracellular content in RORγt^+^ ILC3 (h) and its relative target gene mRNA expression in isolated colonic lymphocytes (i) from VHL^WT^ and VHL^ΔRorc^ mice at steady-state. *N* = 3. (j) body weight variation (left) and clinical scores (right) of infected VHL^WT^ and VHL^ΔRorc^ mice after HBO. *N* = 4. (k-m) colon length (k), absolute number of live CD45^+^CD11b^+^Ly6G^+^ neutrophils in colonic lamina propria (l), and relative bacterial 16S rDNA quantification (m) on day 4 post-infection in HBO-treated VHL^WT^ and VHL^ΔRorc^ mice. *N* = 4. (n) percentage of colonic RORγt^+^ ILC3 (left) and *ex vivo* production of IL-22 and IL-17 (right) from HBO-treated VHL^WT^ and VHL^ΔRorc^ mice. *N* = 4. All mice were littermates and matched by age/sex. Results are representative of at least two independent experiments and presented as mean ± SEM.

Previous reports have shown that butyrate acts through different signaling pathways, including activation of G protein-coupled receptors (GPCRs) and inhibition of histone deacetylase (HDAC).^40^ Furthermore, butyrate stabilizes HIF-1α in intestinal epithelial cells, sustaining their function in homeostasis and in colitis models.^41,44^ HIF-1α forms a heterodimeric transcription factor with the aryl hydrocarbon receptor nuclear translocator (ARNT)^7^. HIF-1α is hydroxylated by prolyl hydroxylases in the presence of oxygen and is subsequently degraded, whereas ARNT is constitutively expressed. Expression of HIF-1α and some of its target genes was reduced in colonic LP lymphocytes isolated from mice exposed to HBO therapy for 5 days (Figure 7a; **S8a**) or HBO-treated mice 4 days after CDI (Figure 7b). Conversely, butyrate supplementation increased the expression of HIF-1α target genes (*Pfkb3*, *Ttf3*, *Scl2a1* and *Ldha*) in colonic LP lymphocytes after CDI (Figure 7b), indicating that butyrate induced HIF-1α activity in LP lymphocytes.

Previous studies have shown that HIF-1α activation regulates ILC3 responses^48–50^and that these cells are important for protecting mice against enteric infection.^35,54^ Next, we hypothesized that butyrate-induced protection during CDI could also be due to the activation of HIF-1α signaling in ILC3. To test this, we first examined the effect of butyrate on CDI in mice lacking HIF-1α in cells expressing RORγt (*Hif-1α*^ΔRorc^), which include ILC3s and T cells (**Fig. S8b**). Butyrate-treated *Hif-1α*^ΔRorc^ mice showed greater weight loss, higher clinical score (Figure 7c), shorter colon (Figure 7d), and greater bacterial translocation (Figure 7e) during CDI than butyrate-treated wild-type mice, indicating that the beneficial effect of butyrate on ILC3 and T cells in CDI is HIF-1α dependent. *Hif-1α*^ΔRorc^ mice also showed a lower frequency of ILC3 (mainly the CCR6^+^ subset) in colonic LP (Figure 7f ; S8c), as well as lower production of IL-22 and IL-17, and higher production of IFNγ (Figure 7g ; S8d). Conditional deletion of *Hif1a* had no effect on the frequency of ILC1 and ILC2 after butyrate treatment (**Fig. S8e**).

In a gain-of-function experiment, we examined the impact of hyperoxia on CDI in mice overexpressing HIF-1α in ILC3 and T cells due to conditional deletion of VHL (Figure 7h, i), an E3-ubiquitin ligase that binds and downregulates HIF-1α and HIF-2α in the presence of oxygen.^51^ These *Vhl*^*ΔRorc*^ mice showed higher levels of ILC3 (of the NCR^−^ subtypes) and increased expression of RORγt, IL-22, and IL-17 in the colonic LP at a steady state (**Fig. S8f-h**). *Vhl*^*ΔRorc*^ mice had milder CDI than WT mice, presenting less weight loss, lower clinical scores, longer colons, less neutrophil accumulation, and bacterial translocation, both in normoxia and after HBO (Figure 7j-m ; S8i,j). In addition, ILC3 were more frequent and produced more IL-22 and IL-17 in the colon 4 days p.i. in *Vhl*^*ΔRorc*^ than in WT mice (Figure 7n ; S8k), confirming the important role of HIF-1α in these cells. The ratio of ILC3 subtypes and frequencies of other ILCs were similar in *Vhl*^*ΔRorc*^ and WT mice during CDI (**Fig. S8l,m**). Although the frequency of RORγt^+^ CD4^+^ T cells in the LP was similar between *Vhl*^*ΔRorc*^ and WT mice, the production of IL-22 and IL-17 by these cells was higher in *Vhl*^*ΔRorc*^ mice (**Fig. S8n**), suggesting that HIF-1/2 may also play a role in T cells responses.

To validate the impact of hyperbaric oxygen and butyrate on ILC3 responses, we conducted a series of *in vitro* experiments. First, incubation of primary ILC3 at high oxygen levels reduced IL-22 production (**Fig. S9a**). Conversely, butyrate treatment maintained the ability of ILC3 to produce more IL-22 (**Fig. S9a**). This *in vitro* effect was related to HIF-1α signaling in an ILC3 murine cell line (MNK3), since IL-22 production was also higher in the presence of HIF-1α and −2α stabilizers (BAY 85–3934), but not using HIF-1α and −2α inhibitors (BAY 87–2243) (**Fig. S9b**). Furthermore, treating primary ILC3 *in vitro* with butyrate increased IL-22 and IL-17 production, but not IFN-γ, in HIF-1α-sufficient ILC3, whereas a less robust response was observed in HIF-1α-deficient cells (**Fig. S9c**). Taken together, our data indicate that butyrate is capable of modulating ILC3 responses through HIF-1 activation, suggesting a mechanism that potentiates the production of IL-22 by these cells and mitigates CDI in mice.

## Discussion

Hyperbaric oxygen therapy (HBO) is used in the treatment of inflammatory diseases. In a randomized controlled trial involving 20 patients with inflammatory bowel disease (IBD), divided into HBO intervention and control groups, HBO treatment demonstrated notable benefits such as increased neovascularization, enhanced intestinal mucosal healing, and improved intestinal motility.^52^ Other studies have indicated that HBO therapy can ameliorate colitis activity in patients with ulcerative colitis (UC) by mitigating neutrophil responses and influencing microbiota composition.^21^ However, it’s crucial to acknowledge that HBO may not be universally effective in treating all patients with UC,^53^ emphasizing the need for additional studies to understand the impact of this treatment on intestinal disorders. In our study, we demonstrated that HBO therapy exacerbates gut dysbiosis and impairs the production of short-chain fatty acids in the intestine, thereby increasing susceptibility to *C. difficile* infection in mice. Concurrently, our findings also revealed that elevated oxygen levels resulting from HBO compromises the host immune responses against *C. difficile*, leading to a reduction in the HIF-1 signaling in ILC3 and a subsequent decrease in IL-22 production, which plays a pivotal role in the context of CDI.^54–57^ Remarkably, administration of the SCFA butyrate can modulate HIF-1 signaling in these cells, exerting protective effects in mice following HBO therapy. This suggests a potential avenue for intervention, wherein the administration of butyrate could mitigate the negative impacts of HBO on gut health and immune responses, offering a promising strategy for safeguarding against enteric infection.

The intestinal microbiota, predominantly composed of the phyla *Firmicutes* and *Bacteroidota*, plays a crucial role in host metabolism and is related to resistance to colonization by enteric pathogens^9^. Qualitative or quantitative changes in the microbiome dramatically affect intestinal mucosal homeostasis. In the present study, we showed that HBO treatment accentuated antibiotic-induced dysbiosis by a significant reduction in the phyla *Verrucomicrobiota*, *Firmicutes*, and *Bacteroidota*, and a drastic expansion of *Proteobacteria*. This observation corroborates previous reports about the increased *Proteobacteria* and *Actinobacteria* in HBO-treated mice.^58,59^ *Proteobacteria* phylum includes a wide variety of pathogenic genera, such as *Escherichia*, *Salmonella*, *Vibrio*, *Helicobacter*, *Yersinia*, and *Legionellales.*^39^ In addition, previous studies have shown that oxygen modulates the composition of the commensal microbial community.^6,8^ The fluctuation between facultative and obligate anaerobes is strongly correlated with the microbiota profile in healthy individuals and those with intestinal diseases.^10^ The reduction of *Firmicutes*, especially those sensitive to oxygen, such as *Faecalibacterium prausnitzii*^60^and *Bacteroidetes*,^61^ is associated with IBD. Similarly, *Akkermansia*, a genus of the phylum *Verrucomicrobia*, which colonizes the gastrointestinal tract and plays a role in mucus degradation and the production of intestinal SCFA, was found to be reduced in mice treated with HBO regardless of antibiotic treatment. *Akkermansia muciniphila* is one of the main species of this genus and has been associated with anti-inflammatory properties, protecting the host against conditions such as type 1 and type 2 diabetes, obesity, and inflammatory bowel disease (IBD).^62–66^ Furthermore, a recent study demonstrated that *A. muciniphila* exerts significant effects on the microbiota community and function, protecting mice from *Clostridioides difficile* infection (CDI).^67^ Thus, the reduction in oxygen-sensitive species that play a crucial role in intestinal homeostasis illustrates the negative impact of HBO on microbiota composition and may be related to the emergence of intestinal disorders.

To test the impact of HBO-induced changes in the microbiota, we used the *C. difficile* model. This bacterium is a spore-forming gram-positive bacillus that is resistant to a wide range of antibiotics, and is one of the most common pathogens associated with antibiotic therapy and nosocomial colitis.^34,68^ We previously demonstrated that lower levels of oxygen in the intestine support host immunity against CDI.^48^ However, there is limited information on how intestinal oxygen fluctuations as well as HBO affect the emergence of enteric infections. A recent study showed COVID-19 patients admitted to the ICU have intestinal dysbiosis resembling that observed after antibiotic therapy and recurrent CDI.^69^ Similarly, an increased prevalence of *C. difficile* and other enteric infections in COVID-19 positive patients receiving oxygen.^70–72^ However, the causal relationship between the increased mortality of these ICU-admitted patients and oxygen or mechanical ventilation is still unclear. In this context, it will be interesting to investigate if SARS-CoV-2 dysbiosis predisposes patients to CDI and whether oxygen administration could be related to worsening dysbiosis in these patients.

It is important to highlight the relationship between increased tissue oxygenation and changes in the gut microbiome after HBO therapy, which affects luminal short-chain fatty acid levels. These metabolites are mainly produced by obligate anaerobes through the fermentation of undigested dietary fiber.^73^ There are numerous reports on their role in controlling host immunity, metabolism, and disease development, as reviewed by our group^40^. SCFAs, particularly butyrate, regulate the expression of several genes relevant to epithelial barrier integrity.^41,43,44^ We have previously reported that butyrate protects against *C. difficile* infection in mice.^41^ Here, we found that HBO induced a more intense reduction in the intestinal concentrations of this metabolite, and that oral supplementation with butyrate was sufficient to attenuate CDI in both groups (normoxia and HBO-treated). These findings are in accordance with those of other studies, indicating the beneficial and anti-inflammatory role of butyrate in colitis models.^74–77^ In addition, a recent study also correlated butyrate with intestinal CD4^+^ T cells and ILCs responses through GPR41 receptor activation and histone deacetylase inhibition.^74^ Butyrate also upregulates the aryl hydrocarbon receptor and HIF-1α in lymphocytes, thus enhancing IL-22 production and intestinal protection against *Citrobacter rodentium* infection.^74^ Other studies have also shown the role of SCFA in controlling the number and function of ILCs in different tissues,^35,46,78^ demonstrating the role of these metabolites in controlling innate lymphocyte responses.

In particular, ILC3 play a crucial role in maintaining mucosal homeostasis and protecting the gut against various pathogens.^23^ IL-22 released by these cells transmits signals through the activation of nuclear factor (NF)-κB and phosphorylation of STAT3 (pSTAT3) in epithelial cells, and controls intestinal stem cell proliferation and differentiation during colitis,^47,79^ as well as the expression of antimicrobial peptides, mucin, cytokines, and chemokines.^25,79^ Studies have already shown that IL-22-deficient mice exhibit gut dysbiosis, increased susceptibility to enteric infections, and unpaired host immune responses.^80^ Gonzalez *et al*.^21^ recently reported that HBO-treated patients showed a significant reduction in both STAT3 and pSTAT3 levels in the neutrophils. Here, we showed that HBO downregulated the production of IL-22 by ILC3, in addition to lowering IL-22 target gene expression by epithelial cells, supporting the idea of an impaired ILC3 response by HBO and their role in maintaining epithelial barrier integrity. Similarly, we also previously showed that intestinal hypoxia upregulates the HIF-1α-IL-22 axis in ILC3 and is associated with increased protection against CDI.^48^

As the gut mucosa is characterized by low oxygen viability at steady state, HIF-1α is commonly active in intestinal epithelial and LP cells.^5,7^ The absence of HIF-1α in the intestinal epithelium aggravates colitis and impairs the epithelial barrier.^41,44^ However, the cell type – specific expression of HIF-1α has been shown to play distinct functions, mainly in the innate compartment. Deletion of HIF-1α in myeloid cells ameliorated experimental colitis,^81^ whereas conditional knockout mice for dendritic cells showed worsened colitis.^82^ Similarly, deletion of HIF-1α impairs neutrophil metabolism, bactericidal activity, and motility.^83^ Here, we showed that deletion of HIF-1α in RORγt^+^ ILC3s impaired murine mucosal defenses after HBO therapy and aggravated *C. difficile* infections. Yang *et al*.^74^ also showed that HIF-1α and AhR mediate butyrate induction of IL-22 in CD4+ T cells, thereby protecting the intestines from *Citrobacter rodentium* infection. In parallel, it has been shown that HIF-1α directly promotes the transcription of the gene encoding RORγt and its targets, such as IL22 and IL17, in humans and mice.^48,74,84^ Moreover, increased glycolysis, OXPHOS, and mitochondrial ROS induced by mTORC-HIF-1α play a crucial role in cytokine secretion and ILC3 proliferation in the intestinal lamina propria.^48,49^

By contrast, a recent study by Krzywinska *et al*.^85^ showed that mice lacking the HIF-1α isoform in NKp46^+^ ILC3 have increased IL-22 production and a protective phenotype during MTX- and DSS-induced colitis. Some explanations could justify the discrepancy between the models. First, the RORγt promoter used in our study deleted HIF-1α simultaneously in NKp46^−^ and NKp46^+^ ILC3s, as well as partially in CD4^+^ T cells, although these cells have not been shown to be relevant in the murine model of CDI.^54^ However, conditional deletion of HIF-1α using NCR-Cre mice, as in Krzywinska *et al*.^85^ affects NKs and ILC1 and only becomes effective for ILC3 after the acquisition of an ILC1-like phenotype, the NCR^+^ ILC3 subset.^86–88^ The effect of HIF-1α on ILC1 remains unclear, although natural killer cells have been shown to be affected.^89^ Second, the different spatial distributions of NKp46^+^ and NKp46^−^ ILC3 in the intestinal mucosa, as well as the differences in oxygen gradients, could impact the relevance of HIF-1α in the activation of these cells, similar to that shown before for CXCR5 in CD4^+^ ILC3.^90^ Finally, although different deletion strategies target HIF-1α at different stages of ILC development, the colitis models studied here also provide different metabolic conditions along the host mucosa. In the future, strategies for better deletion of HIF-1α in specific ILC subtypes, mainly ILC1 and ILC3 subtypes, and ILC progenitors would provide more information about the role of HIF-1α in the maintenance of innate lymphoid responses. Likewise, fate map analysis could also add information about the plasticity of these cells, which depends on the microenvironment and/or HIF-1α activity.

One notable limitation in our study arises from the exclusive utilization of *C. difficile* vegetative bacteria, as oral spore ingestion represents a more natural mode of transmission for this infection.^91^ Spores are highly resistant to environmental challenges, including disinfectants and extreme temperatures, thus allowing them to persist in the environment for extended periods. Investigations involving spores prove crucial for understanding the transmission dynamics and environmental persistence of the bacterium. On the other hand, research with vegetative bacteria explores infection pathogenesis and host immune responses. Both approaches, with their distinct emphases, collectively contribute to our comprehension of *C. difficile* infection.^92^ After oral ingestion and upon reaching the colon, the spores undergo germination, transitioning into the active, vegetative form that leads to *C. difficile* colonization and toxins release^93,94^ The gut microbiota provides resistance against colonization by *C. difficile* and other opportunistic pathogens.^95^ Bile acid metabolites are relevant in this context.^96^ While primary bile acids, especially taurocholate, activate *C. difficile* spore germination, secondary bile acids exert the opposite effect.^96^ Alterations in the microbiota induced by antibiotics or, as demonstrated in this study, by HBO, can influence bile acid metabolism,^21^ potentially impacting on spore germination and *C. difficile* colonization. These aspects need to be investigated in future studies.

Importantly, our study did not assess the application of HBO in treating mice with CDI; rather, we elucidated mechanisms by which HBO influences the development of CDI in mice. Conventional CDI management involves antibiotics treatment, like metronidazole, vancomycin, or fidaxomicin, targeting and eliminating *C. difficile.*^97^ In contrast, HBO is a well-established modality used in treating chronic non-healing wounds and certain type of infections. A recent retrospective study revealed potential benefits of HBO for patients with *C. difficile* infection, by shortening symptomatic periods and preventing recurrence.^98^ Although HBO is not considered a standard practice for CDI treatment, future research may shed light on its role in managing *C. difficile* infection, contributing to a more comprehensive understanding of its therapeutic potential for enteric infection.

Taken together, we showed that HBO increases gut dysbiosis, which is associated with ILC3 impairment and an increased susceptibility to *C. difficile* infection. Treatment with microbiota-derived butyrate during HBO significantly reduced inflammation and enhanced the epithelial barrier integrity. We substantiated the pivotal role of butyrate in regulating ILC3 responses through the targeted manipulation of HIF-1α in RORγt+ cells. In the future, translational studies are necessary to ascertain SCFAs levels in HBO patients and to check the potential of enhancing butyrate production as a therapeutic strategy for palliative treatment. Targeting the modulation of HIF-1α in the intestine represents a promising therapeutic approach for managing intestinal inflammation.

## Material and methods

### Mice

C57BL/6 male mice were purchased from the Multidisciplinary Center for Biological Investigation, Campinas-SP, Brazil. RORγt-Cre^+^, HIF-1alfa^floxed/floxed^, and VhL^floxed/floxed^ mice were obtained from Jackson Laboratories. All strains were maintained on a C57BL/6 background and kept in regular filter-top cages with free access to sterile water and food. Animal procedures were approved by the Ethics Committee on Animal Use of the University of Campinas (protocols # 5730–1/2021 and 6252–1/2023).

### Hyperbaric oxygen (HBO) treatment

For *in vivo* administration of hyperbaric oxygen, 7–8-week-old mice were exposed for 90 min daily for 5 days to 100% oxygen at a pressure of 2.5 atmospheres (ATA) in a hyperbaric animal research chamber (Model HB 1300 B; Sechrist, Anaheim, CA, USA), according to a previously described protocol.^99,100^ The chamber was pressurized and decompressed at a rate of 0.5 ATA/min. Control mice were kept in a ventilated room with normal oxygen tension and local atmospheric pressure (0.98 ATA).

### *C. difficile* infection

*Clostridioides difficile* VPI 10,463 and PCR ribotype 078 strains were cultivated in BHI blood agar at 37°C in anaerobiosis (AnaeroGen, Oxoid; Thermo Fisher Scientific). Eight-week-old age- and sex-matched mice were pre-treated with an antibiotic mixture (0.4 mg/mL kanamycin, 0.035 mg/mL gentamicin, 0.035 mg/mL colistin, 0.215 mg/mL metronidazole, and 0.045 mg/mL vancomycin; Sigma) for 4 days through drinking water supplementation, as previously described.^33,41^ Antibiotics were discontinued, and mice received a single intraperitoneal dose of clindamycin (10 mg/kg) (Sigma). One day later, the mice were infected with 1 × 10^8^ colony forming units (CFUs) of *C. difficile* by gavage. They were weighed daily and monitored for clinical severity scores, which varied from 0 (normal) to 15 (dead) (**Table S1**).

### SCFA butyrate treatment

Butyrate 150 mM had a pH adjusted to 7.2 and was added to drinking water for the oral treatment of mice, as reported in other studies.^41,101^ The treatment started one day before the addition of antibiotics (day −7) and continued throughout the end of the experiments. The control group received normal water without SCFA supplementation.

### Quantitative gene expression

Total RNA was extracted from the tissues using a PureLink^TM^ RNA kit (Ambion). RNA was converted to cDNA using the High-Capacity cDNA Reverse Transcription Kit (Applied Biosystems), and qPCR was performed using Power SYBR Green PCR Master Mix (Applied Biosystems) and the primers listed in **Table S2**. Gene expression was quantified using the 2^ΔΔ^Ct method with β2-microglobulin as a reference gene.

### Intestinal lamina propria cells isolation

The small intestine or colon was harvested from mice, cut longitudinally, and washed twice with Hanks’ Balanced Salt solution (Thermofisher) to remove the luminal content. Intraepithelial lymphocytes (IELs) were excluded by two 20-minutes washes with room-temperature HBSS/HEPES +5 mM EDTA + 10% bovine serum. The tissues were then placed in a 37°C shaker for 40 min with 1 mg/mL collagenase IV (Sigma). Immune cells were enriched over a 40%/70% Percoll gradient (GE Healthcare), washed, stimulated *ex vivo* and/or labeled with monoclonal antibodies for analysis by flow cytometry (**Table S3**).

### Flow cytometry

For FACS analysis, dead single-cell preparations of the small intestine or colon lamina propria were excluded using a live/dead cell viability assay in the Brilliant Violet 510. A lineage cocktail containing R-phycoerythrin-conjugated monoclonal antibodies against CD3, CD5, and CD19 was used, except where stated, and ILC were identified as Lin^−^ and CD45^+^ (PE-Cy7). Surface staining was performed with antibodies diluted in FACS buffer at 4°C for 20 min in the dark after blocking the Fc receptors with purified anti-CD16/CD32 (BioLegend). When needed, cells were fixed and intracellularly stained using the Foxp3 Staining Buffer Set (eBioscience), according to the manufacturer’s instructions. For functional experiments, MNK3 or primary cells were cultured in 96-well plates with complete media and stimulated with cytokines and/or Golgi Plug (BD Biosciences) for 3 hours at 37°C. Following incubation, Live^+^ Lin^–^ (CD3^–^CD5^–^CD19^–^) CD45^int^CD90.2^high^ILC3s were stained for viability, surface molecules, fixed in 2% paraformaldehyde (PFA), and intracellularly stained using the BD Biosciences Fixation/Permeabilization Solution Kit (**Table S3**). Samples were acquired using BD FACS-Symphony™ with BD FACSDiva™ Software (BD Biosciences). All FACS data were analyzed using FlowJo v.9.5.2 software (Tree Star).

### Red blood cells analysis

The mice were anesthetized with ketamine/xylazine (85 mg/kg, 15 mg/kg i. p.). Blood samples were collected via cardiac puncture into heparin-containing tubes, and cell counts were performed using an automated hematocytometer counter (Beckman Coulter AcT Diff Hematology Analyzer, Brea, California, USA).

### Epithelial permeability assay

Food and water were removed for 4 h, and then mice received 250 mg/kg of FITC-dextran (70,000 Da; Sigma) suspension in PBS by gavage. After 4 h, the rats were anesthetized, and blood was collected by cardiac puncture. FITC-dextran fluorescence was quantified using a Multi-Mode Microplate Reader (Synergy HT; Vermont, USA) at 485 nm excitation and 528 nm emission wavelengths. A standard curve was prepared using serial dilutions of 100 µg/mL FITC-dextran in phosphate-buffered saline (PBS).

### Bacterial translocation

The spleen, liver, and mesenteric lymph nodes were harvested from *C. difficile* infected mice for the analysis of intestinal bacterial translocation. Bacterial 16S rDNA was extracted using the PureLink^TM^ Microbiome DNA Purification kit (ThermoFisher Scientific), and gene levels were quantified by qPCR using primers complementary to the Eubacteria 16S rDNA conserved gene (**Table S2**). The bacterial load was determined using an *E. coli* genomic DNA standard curve, and then the relative number of 16S rDNA gene copies were normalized by the sample weight.

### C. difficile CFU counts

Stool samples harvested on days 2 or 5 post-infection were weighed, vortexed in 1 mL sterile PBS, and left for 10 min prior to decantation. Supernatants were diluted at 10^−–6^ and 10^−–7^ and plated on cycloserine-cefoxitin-fructose-agar supplemented with horse blood. The plates were incubated in an anaerobic atmosphere at 37°C for 4–5 days.

### C. difficile toxin TcdA/TcdB quantification

Toxins were measured in stool samples using a Ridascreen® *C. difficile* Toxin TcdA/TcdB ELISA kit (R-Biopharm, Darmstadt, Germany). Samples were harvested on days 2 or 5 post-infection, weighed, and vortexed in 1 mL dilution buffer. Samples were left for 10 min prior to decanting, and then the supernatant was collected for quantification, following the manufacturer’s recommendations.

### Quantification of cytokines by ELISA

Approximately 100 mg of colon tissue was harvested and homogenized in PBS containing protease inhibitors (Thermo Fisher Scientific). Samples were centrifuged for 10 min at 2000 × g, and supernatants were used for quantifying TNF-α, IL-6, IL-1β, IL-10, KC (Cxcl1), and MIP-2 (Cxcl2) using the Duo Set ELISA kit (R&D Systems; Minneapolis, MN, USA). Data were normalized to the total protein concentration determined using the Bradford protein assay.

### Microbiota metataxonomic analysis

Fecal samples were collected in sterile DNA-free tubes and frozen in liquid nitrogen. DNA extraction was performed using the Purelink Microbiome DNA Purification Kit (Thermo Fisher Scientific) following the manufacturer’s recommendations. Universal primers 341F (5′- AYG GGR BGC ASC AG-3) and 806 R (5′- CTA CNN GGG TAT CTA AT-3) were used to amplify the V3-V4 region of the bacterial 16S rRNA gene. Library quantification and quality were assessed using a Qubit 2.0 Fluorometer (Thermo Scientific) and Agilent Bioanalyzer 2100 systems. Libraries were prepared and sequenced (PE250) on a NovaSeq 6000 Sequencing System (Illumina, USA) by Novogene (China).

Quality control of raw FASTQ files was performed using FastQC and MultiQC.^102^ The sequence files were then imported into QIIME2 version 2021.11,^103^ and processed for removal of noisy, chimeric sequences and singletons, joining of paired-end reads, dereplication, and obtainment of amplicon sequence variants (ASVs) using the DADA2 plugin.^104^ A phylogenetic tree was constructed using QIIME2’s fragment-insertion SEPP plugin with the Silva 128 reference database, and the taxonomic composition was assessed with QIIME2’s q2-feature-classifier plugin using a Naive Bayes classifier pre-trained on Silva 138 99% OTUs full-length sequences.^104,105^

QIIME2 resulting files were imported into R using the phyloseq package (McMurdie, 2013). Taxa bar plots and diversity analyses were performed using the microViz R package (Barnett 2021). Alpha diversity was calculated as the Shannon index, whereas the beta diversity metric consisted of Bray-Curtis/Unweighted UniFrac distance. Differential analysis was conducted with the R package microbiomeMarker^106^using the ANOVA-Like Differential Expression tool (ALDEx2)^107^ and linear discriminant analysis Effect Size (LEfSe).^107^ Functional abundances were predicted using Phylogenetic Investigation of Communities by Reconstruction of Unobserved States (PICRUSt2)^108^ and visualized using the R package ggpicrust2.^109^ 16S sequencing data were deposited in the NCBI BioProject: PRJNA900580.

### SCFA quantification by gas chromatography (GC)

The proximal colon content was harvested from the mice as previously described.^101^ Chromatographic analyses were performed using a GC-QP2010 Ultra mass spectrometer (Shimadzu; ThermoFisher Scientific) and a 30 m^3^ 0.25 mm fused-silica capillary Stabil wax column (Restek Corporation, Bellefonte, PA, USA) coated with 0.25-mm polyethylene glycol. The samples were injected at 250°C at a 25:1 split ratio. High-grade pure helium was used as the carrier gas at 1.0 a constant flow. The mass conditions were as follows: ionization voltage, 70 eV; ion source temperature, 200°C; full scan mode, 35–500 m/z with 0.2 a scan velocity. The runtime was 11.95 min.

### Histopathological analysis

Mouse colons were harvested, opened longitudinally, and fixed in 4% formalin/0.1% glutaraldehyde. Tissues were processed into paraffin and 5-µm sections were prepared for staining with hematoxylin and eosin (H&E) solution and periodic acid – Schiff (PAS) stain kit. Images were obtained using an U-LH100HG Olympus Microscope. Histopathological analysis was performed using H&E-stained sections, and tissue inflammation was determined by a total score from 0 to 30, which represents the sum of 10 parameters evaluated from 0 (normal) to 3 (severe) (**Table S4**). The frequency of goblet cells and epithelial mucin content were quantified in PAS sections using a score from 0 to 3 (**Table S5**).

### Fluorescent in situ hybridization (FISH)

FISH was performed as previously described by Molloy et al.^110^ Colon fragments were fixed in methacarn for 3 h at 4°C and 5 µm coronal slices were obtained. Sections were incubated with 100 nM bacterial probes (GCTGCCTCCCGTAGGAGT; FITC-conjugated; Sigma), 20 mg/mL lectin-Ulex europaeus agglutinin-I (UEA-I; Tetramethyl-rhodamine-conjugated, Sigma), 10 mg/mL Hoechst 33,258 solution, and mounted with SlowFade Gold medium (Thermo Fisher Scientific). Images were acquired using a Zeiss LSM-780 confocal microscope (Carl Zeiss, Oberkochen, Germany). Samples were imaged with a 63×/1.4NA oil-immersion objective at 3× with a 1024 3 1024 frame size.

### *In vitro* assay

Primary small intestine ILC3 or MNK3 cell lines, previously described as an *in vitro* system to study ILC3 functionality,^111^ were cultured in complete RPMI (Corning) containing 10% fetal bovine serum, 2 mM GlutaMAX, 1 mM sodium pyruvate, 55 μM 2-mercaptoethanol, and 50 μg/mL gentamicin (ThermoFisher). Conditioned medium containing IL-2 and IL-7 was also used to maintain MNK3 cells. For the hyperoxygenation assays, 2 × 10^5^ cells were cultured in 96 round-bottomed wells polystyrene plates (Corning), placed in a chamber with 100% oxygen injection for 15 min, and cultivated for 3 h at 37°C. The normoxic controls were incubated for the same period in 5% CO_2_ at 37°C. Cells were also treated with IL-1β (10 ng/mL), IL-23 (10 ng/mL), Bay 87–2243/HIF-1/2 inhibitor (10 µM, Sigma-Aldrich), Bay 85–3934/HIF-1/2α stabilizer (10 µM, Sigma-Aldrich), and brefeldin A (BD Golgi Plug, 1:1000).

### Statistical analysis

Analyses were performed using the GraphPad software (version 8.0; San Diego, CA, USA). Experiments were repeated at least twice, and the data are presented as the mean ± SEM. Differences between samples were considered significant at *p* < 0.05. Results were compared using Student’s t-test or Mann – Whitney U test, as appropriate. For more than two groups, differences were compared using one-way analysis of variance (ANOVA), followed by Tukey’s *post hoc* test.

## Supplementary Material

Supplemental Material

## Data Availability

All the relevant data supporting these findings are available in this report. In particular, 16S sequencing data have been deposited in the NCBI BioProject: PRJNA900580.
