# Supplementary material for: Hyperbaric oxygen augments susceptibility to C. difficile infection by impairing gut microbiota ability to stimulate the HIF-1α-IL-22 axis in ILC3
Source: Gut Microbes. 2024 Jan 2;16(1):2297872. doi: 10.1080/19490976.2023.2297872 (PMC10763646; doi:10.1080/19490976.2023.2297872)
Supplement: Supplemental Material [file KGMI_A_2297872_SM0505.zip › supp table and fig captions.docx]

**SI Appendix**

**Table S1.** Clinical score for *C. difficile* infection.

| **Category** | **Score*** |  |  |  |
| --- | --- | --- | --- | --- |
|  | **0** | **1** | **2** | **3** |
| **Activity** | Normal | Alert/Slow moving | Lethargic/Shaky | Inactive unless prodded |
| **Posture** | Normal | Back slanted | Hunched | Hunched/Nose down |
| **Coat** | Normal | Piloerection | Rough skin | Very ruffled Puff/Ungroomed |
| **Diarrhea** | Normal | Soft stool/Discolored (yellowish) | Wet stained tail/ mucous +/- blood | Liquid/no stool (ileus) |
| **Eyes/Nose** | Normal | Squinted  ^1^/_2_ closed | Squinted/Discharge | Closed/Discharge |

*Clinical score = sum of all parameter scores. The total possible score was 15 (deaths).

**Table S2.** qPCR primers.

| **Primer** | | **Sequence (5’ – 3’)** |
| --- | --- | --- |
| ***Eubacteria* (rDNA 16S)** | Forward | ACT CCT ACG GGA GGC AGC AGT |
|  | Reverse | ATT ACC GCG GCT GCT GGC |
| ***B2m*** | Forward | CCC CAC TGA GAC TGA TAC ATA CG |
|  | Reverse | CGA TCC CAG TAG ACG GTC TTG |
| ***Il17*** | Forward | TCA GCG TGT CCA AAC ACT GAG |
|  | Reverse | GAC TTT GAG GTT GAC CTT CAC AT |
| ***Il22*** | Forward | AGA ATG TCA GAA GGC TGA AGG |
|  | Reverse | AGG AGC AGT TCT TCG TTT TCT AG |
| ***Rorc*** | Forward | TCC ACT ACG GGG TTA TCA CCT |
|  | Reverse | AGT AGG CCA CAT TAC ACT GCT |
| ***Ldha*** | Forward | ACG CAG ACA AGG AGC AGT GGA A |
|  | Reverse | ATG CTC TCA GCC AAG TCT GCC A |
| ***Slc2a1*** | Forward | CTT TGT GGC CTT CTT TGA AGT |
|  | Reverse | CCA CAC AGT TGC TCC ACA T |
| ***Pfkfb3*** | Forward | GGA GGT CGG CAT GTT GAA GA |
|  | Reverse | CTT TGG AAG GGC CTG AGA GG |
| ***Tff3*** | Forward | TGC AGA TTA CGT TGG CCT GT |
|  | Reverse | TGC AGA GGT TTG AAG CAC CA |
| ***Hif1a*** | Forward | ATC TCG GCG AAG CAA AGA GTC |
|  | Reverse | TGG GGA AGT GGC AAC TGA T |
| ***Tbx21*** | Forward | AAC CGC TTA TAT GTC CAC CCA |
|  | Reverse | CTT GTT GTT GGT GAG CTT TAG C |
| ***Foxp3*** | Forward | ACC ATT GGT TTA CTC GCA TGT |
|  | Reverse | TCC ACT CGC ACA AAG CAC TT |
| ***Muc1*** | Forward | CCC TAC CTA CCA CAC TCA CGG ACG |
|  | Reverse | GTG GTC ACC ACA GCT GGG TTG GT |
| ***Muc2*** | Forward | CGA CTG TGA GCA GTG TGT CA |
|  | Reverse | GGG TAG GGT CAC CTC CAT CT |
| ***Muc4*** | Forward | GAG GGC TAC TGT CAC AAT GGA GGC |
|  | Reverse | AGG GTT CCG AAG AGG ATC CCG TAG |
| ***Reg3g*** | Forward | TTC CTG TCC TCC ATG ATC AAA A |
|  | Reverse | CAT CCA CCT CTG TTG GGT TCA |
| ***Defb1*** | Forward | CCA GAT GGA GCC AGG TGT TG |
|  | Reverse | CTG GAG CGG AGA CAG AAT CC |
| ***Defb3*** | Forward | GCA TTG GCA ACA CTC GTC AGA |
|  | Reverse | CGG GAT CTT GGT CTT CTC TA |
| ***Camp*** | Forward | TCTCTACCGTCTCCTGGACCTG |
|  | Reverse | CCACATACAGTCTCCTTCACT |
| ***Cxcl1*** | Forward | ACT GCA CCC AAA CCG AAG TC |
|  | Reverse | TGG GGA CAC CTT TTA GCA TCT T |
| ***Il1b*** | Forward | GGC AGC TAC CTG TGT CTT TCC C |
|  | Reverse | ATA TGG GTC CGA CAG CAC GAG |
| ***Il16*** | Forward | CTG CAA GAG ACT TCC ATC CAG |
|  | Reverse | AGT GGT ATA GAC AGG TCT GTT GG |
| ***Il2*** | Forward | CCT GAG CAG GGA GAA TTA CA |
|  | Reverse | TCC AGA ACA TGC CGC AGA |
| ***Il10*** | Forward | TGC CAA GCC TTA TCG GAA ATG |
|  | Reverse | AAA TCG ATG ACA GCG CCT CAG |
| ***Tnfa*** | Forward | TCT TCT CAT TCC TGC TTG TGG C |
|  | Reverse | CAC TGG TGG TTT GCT ACG ACG |
| ***Ifng*** | Forward | ATG AAC GCA CAC ACT GCA TC |
|  | Reverse | CCA TCC TTT TGC CAG TTC CTC |

**Table S3.** Antibodies.

| **ANTIBODY** | **SOURCE** | **CLONE** | **IDENTIFIER** |
| --- | --- | --- | --- |
| Anti-mouse CD45, PE/Cy7 | eBioscience | 30-F11 | Cat# 25-0451-82 |
| Anti-mouse CD4, FITC | BioLegend | RM4-4. | Cat# 100510 |
| Anti-mouse CD3, PE | BioLegend | 17A2 | Cat# 100206 |
| Anti-mouse CD5, PE | BioLegend | 53-7.3 | Cat# 100608 |
| Anti-mouse CD19, PE | BioLegend | 6D5 | Cat# 115508 |
| Anti-mouse CD11b, PE | BioLegend | M1/70 | Cat# 101208 |
| Anti-mouse CD11c, PE-Cy7 | BioLegend | N418 | Cat# 117317 |
| Anti-mouse Ly6G, FITC | BioLegend | 1A8 | Cat# 127605 |
| Anti-mouse F4/80, APC | BioLegend | BM8 | Cat# 123116 |
| Anti-mouse CD45 APC/Cy7 | BioLegend | 30-F11 | Cat# 103116 |
| Anti-mouse CD90.2 (Thy-1.2) FITC | BioLegend | 53-2.1 | Cat# 140303 |
| Anti-mouse CD90.2 (Thy-1.2) Brilliant Violet 785 | BioLegend | 30-H12 | Cat# 105331 |
| Anti-mouse CD335 (NKp46), Brilliant Violet 421 | BioLegend | 29A1.4 | Cat# 137612 |
| Anti-mouse CD196 (CCR6), PerCP/Cy5.5 | BioLegend | 29-2L17 | Cat# 129809 |
| Anti-mouse RORγt, PerCP/Cy5.5 | BD Pharmingen | Q31-378 | Cat# 562683 |
| Anti-mouse GATA3, Alexa Fluor-488 | BioLegend | 16E10A23 | Cat# 653808 |
| Anti-mouse Tbet, APC | Biolegend | 4B10 | Cat# 644814 |
| Anti-mouse Foxp3, PE | Biolegend | MF-14 | Cat# 126403 |
| Anti-mouse EOMES, PE-Cy7 | eBioscience | Dan11mag | Cat# 25-4875-82 |
| Anti-mouse IL17, APC-Cy7 | BD Pharmingen | TC11-18H10 | Cat# 560821 |
| Anti-mouse IL22 APC | BioLegend | Poly5164 | Cat# 516409 |
| Anti-mouse IFNy, Brilliant Violet 421 | BioLegend | XMG1.2 | Cat# 505830 |
| Anti-TNFα, PE | BioLegend | MP6-XT22 | Cat# 506305 |
| Anti-human/mouse HIF-1α, Alexa Fluor® 488 | R&D System | 241812 | Cat# IC1935G |
| Anti-human/mouse HIF-1α | Abcam | mgc3 | Cat# ab16066 |
| Anti-mouse IgG1, FITC | eBioscience | M1-14D12 | Ca# 11-4015-82 |
| Anti-mouse Ki67, PerCP/Cy5.5 | BD Pharmingen | B56 (RUO) | Cat# 561284 |
| Live/Dead™ Fixable Aqua Dead Cell Stain Kit, BV510 | Invitrogen | - | Cat# L34957 |

**Table S4.** Histopathological score.

| **Category** |  | **Score*** | | | |
| --- | --- | --- | --- | --- | --- |
|  |  | **0 (none)** | **1 (mild)** | **2 (moderate)** | **3 (severe)** |
| **Mucosal epithelium** | Ulceration | None | Mild surface | Moderate | Extensive full  thickness |
| **Crypts** | Mitotic Activity | Lower  third | Mild mid  third | Moderate mil  third | Upper third |
|  | Mucus depletion | None | Mild | Moderate | Severe |
| **Lamina propria** | Mononuclear  infiltrate | None | Mild | Moderate | Severe |
|  | Granulocyte infiltrate | None | Mild | Moderate | Severe |
|  | Vascularity | None | Mild | Moderate | Severe |
|  | Fibrin deposition | None | Mucosal | Submucosal | Transmural |
| **Sub-mucosal** | Mononuclear  infiltrate | None | Mild | Moderate | Severe |
|  | Granulocyte infiltrate | None | Mild | Moderate | Severe |
|  | Edema | None | Mild | Moderate | Severe |

**Table S5.** Goblet cells score for imaging quantification.

| **SCORE** | **DESCRIPTION** |
| --- | --- |
| **0** | Absent alcian blue staining characterizing complete loss of goblet cells. |
| **1** | Faint alcian blue staining characterizing marked to moderate multifocal to diffuse loss of goblet cells associated with low mucin content in the goblet cells still present. |
| **2** | Moderate alcian blue staining characterizing mild multifocal to diffuse loss of goblet cells associated with intermediate mucin content in the goblet cells still present. |
| **3** | Intense alcian blue staining characterizing adequate number of goblet cells associated with adequate mucin content in the goblet cells present (normal colonic mucosa). |

**Supplementary figures**

**Fig. S1 - Impacts of HBO therapy on the intestinal mucosa.**

**(a)** Body weight variation of mice at different time points after HBO treatment. N = 5. **(b)** Analysis of CCR6^+^, NKp46^+^, and double-negative (DN) RORγt^+^ ILC3 subsets by flow cytometry. N = 5. **(c)** Gating strategy for the identification of innate lymphoid cells (top) and frequency of ILC1 and ILC2 in colonic LP at different time points after HBO (bottom). Live cells were pre-gated on lineage negative (CD3, CD5, CD19) and CD45 positive. ILC1 is GATA3^-^ CD45^high^ NKp46^+^. ILC2 is GATA3^+^. ILC3 is CD45^int^ CD90.2^+^ RORγt^+^. N = 5. **(d)** Gating strategy for the identification of intestinal T cell populations. **(e,f)** Percentage of T cells (e) and IL-22, IL-17, and TNF-α production by CD4^+^ T cells (f) in the colonic LP at different time points after HBO therapy. N = 5. **(g)** Red blood cells distribution width (left) and mean corpuscular hemoglobin (right) analysis by hemocytometry at different time points after HBO. N = 5. All results are from at least two independent experiments and presented as mean ± SEM.

**Fig. S2 – HBO therapy affects the gut microbiome composition.**

**(a,b)** Analysis of gut microbiome β-diversity based on Weighted (a) and Unweighted (b) UniFrac distances of normoxic or HBO-treated mice. N = 5. **(c)** Analysis of gut microbiome α-diversity based on Shannon index. **(d,e)** Bar plots of 16S rDNA gene reads assigned to taxonomy on the bacterial phylum (d) and genus (e) level. (**f**) Differential abundance analysis by ANOVA-Like Differential Expression (ALDEx2). **(g)** Linear discriminant analysis Effect Size (LEfSe) visualized by the abundance.

**Fig. S3 - Analysis of mice susceptibility to *C. difficile* strain RT078 infection.**

**(a,d)** Probability of survival (a), body weight variation (b), and mean clinical score (c) of HBO-treated mice infected with C. difficile strain PCR ribotype 078. Infections were performed using 10^8^ CFU per mouse, after antibiotic treatment. N = 5. **(d)** Mean clinical score at day 7 p.i. N = 5. **(e)** *C. difficile* CFU counts on day 2 (left) and 5 (right) post-infection. N = 5. Results are presented as mean ± SEM.

**Fig. S4 - Analysis of intestinal immunity in HBO-treated mice during CDI.**

**(a)** Gating strategy (left) and frequency of CD11b^+^ F4/80^+^ macrophages (middle) and CD11b^+^ CD11c^+^ dendritic cells (right) in the colonic LP of infected HBO-treated mice. N = 4. **(b)** Cytokines relative mRNA expression in the proximal colon on day 4 p.i. N = 6. **(c,d)** Absolute number of ILC1 (c) and ILC2 (d) in the colon (left) and small intestine (right) lamina propria. N = 3-4. **(e)** Absolute number of CCR6^+^, NKp46^+^ (NCR), and double-negative RORγt^+^ ILC3s in the colon (top) and small intestine (bottom) of HBO-treated mice on day 4 p.i. N = 3-4. **(f)** Absolute number of ILC1, ILC2, and ILC3 in the mesenteric lymph nodes (mLNs) of HBO-treated mice on day 4 p.i. N = 5. **(g,h)** Percentage of IL-17 (g) and IFN-γ (h) content in ILC3 after IL-1β/IL-23 *ex vivo* stimulation. N = 3. All results are from at least two independent experiments and are presented as mean ± SEM.

**Fig. S5 - Analysis of T cells in HBO-treated mice during CDI.**

**(a-c)** Absolute number of CD4^+^ (left) and CD4^+^ RORγt^+^ (right) T cells in the small intestine LP (a), mesenteric lymph nodes (b), and spleen (c) of HBO-treated mice on day 4 post-infection. N = 5. All results are from at least two independent experiments and are presented as mean ± SEM.

**Fig. S2 – HBO exacerbates the gut microbiome dysbiosis after antibiotics.**

**(a,b)** Analysis of microbiome β-diversity based on Weighted (a) and Unweighted (b) UniFrac distances in normoxic or HBO-treated mice after antibiotics treatment. N = 5. **(c)** Analysis of microbiome α-diversity based on Shannon index. **(d,e)** Bar plots of 16S rDNA gene reads assigned to taxonomy on the bacterial phylum (d) and genus (e) level. (**f**) Differential abundance analysis by ANOVA-Like Differential Expression (ALDEx2). **(g)** Linear discriminant analysis Effect Size (LEfSe) visualized by the abundance.

**Fig. S7 - Effects of butyrate supplementation in infected HBO-treated mice.**

**(a,b)** Small intestine (a) and cecum (b) length on day 4 post-infection of butyrate and HBO-treated mice. N = 8-9. **(c)** *C. difficile* toxin A/B luminal concentration quantified by ELISA. N= 5. **(d,e)** Quantification of goblet cells density by scoring criteria (d) and representative histological sections of colon stained with Alcian-blue (e). Scale bars = 50 µm. N = 6. **(f)** Absolute number of CD11b^+^ F4/80^+^ macrophages (left) and CD11b^+^ CD11c^+^ dendritic cells (right) in the colonic LP. N = 8-9. **(g)** TNF-α (left) and IL-6 (right) quantification in proximal colon on day 4 p.i. by ELISA. N = 8-9. **(h,i)** Number of CCR6^+^, NKp46^+^ (NCR), and double-negative RORγt^+^ ILC3s in the colon (h) and small intestine (i) of butyrate and HBO-treated mice on day 4 p.i. N = 3. **(j,k)** Number of ILC1 (left) and ILC2 (right) in the colon (j) and small intestine (k) LP. N = 8-9; 6. All results are from at least two independent experiments and are presented as mean ± SEM.

**Fig. S8 - HIF-1α signaling pathway in HBO-treated mice during CDI.**

**(a)** Relative mRNA expression of HIF-1 target genes at different time points in Percoll-purified colonic lymphocytes. N = 5. **(b-e)** Percentage of HIF-1α^+^ RORγt^+^ ILC3 (b), CCR6^+^, NKp46^+^ (NCR), and double-negative RORγt^+^ ILC3s (c), IFNγ-producing ILC3 (d), and absolute number of ILC1 and ILC2 (e) in the colonic LP of butyrate-treated HIF-1α^WT^ and HIF-1α^ΔRorc^ mice after HBO on day 4 p.i. N = 4. **(f-h)** Percentage of RORγt^+^ ILC3 (f, left) and subsets (f, right), and IL-22/IL-17/RORγt expression in ILC3 by FACS (g) or qPCR (h) at steady state in colonic LP of VHL^WT^ and VHL^ΔRorc^ mice. N = 3. **(i)** Analysis of body weight variation (left) and clinical score (right) during CDI. N = 5. **(j,k)** Percentage of infiltrating Ly6G^+^ neutrophils (j), RORγt^+^ ILC3 and IL-22/IL-17 producing ILC3 (k) in the colonic LP of VHL^WT^ and VHL^ΔRorc^ mice on day 4 p.i. N = 5. **(l)** Frequency of CCR6^+^, NKp46^+^ (NCR), and double-negative RORγt^+^ ILC3s in the colon of 4-day post-infected VHL^WT^ and VHL^ΔRorc^ mice under normoxia (left) or after HBO therapy (right). N = 4. **(m,n)** Percentage of ILCs (m), and RORγt^+^, IL-22^+^, and IL17^+^ CD4^+^ T cells (n) in the colonic LP of HBO-treated VHL^WT^ and VHL^ΔRorc^ mice on day 4 p.i. N = 5. All results are from at least two independent experiments and presented as mean ± SEM.

**Fig. S9 - Butyrate mediates *in vitro* HIF-1α signaling in ILC3.**

**(a,b)** Percentage of IL-22 producing RORγt^+^ ILC3 (a) or ILC3 cell line MNK3 (b) after *in vitro* stimulation. Us = unstimulated cells; St = IL-1β/IL-23 stimulation; Bt = butyrate; Stab = HIF-1α activator; Inhib = HIF-1α inhibitor. N = 5-6. **(c)** IL-22, IL-17 and IFNγ production by RORγt^+^ si-ILC3 from HIF-1α^WT^ and HIF-1α^ΔRorc^ mice. N = 3. Results are representative of two independent experiments and presented as mean ± SEM.
