# Supplementary figures and images for: Hyperbaric oxygen augments susceptibility to C. difficile infection by impairing gut microbiota ability to stimulate the HIF-1α-IL-22 axis in ILC3
Source: Gut Microbes. 2024 Jan 2;16(1):2297872. doi: 10.1080/19490976.2023.2297872 (PMC10763646; doi:10.1080/19490976.2023.2297872)

**FIG S1**


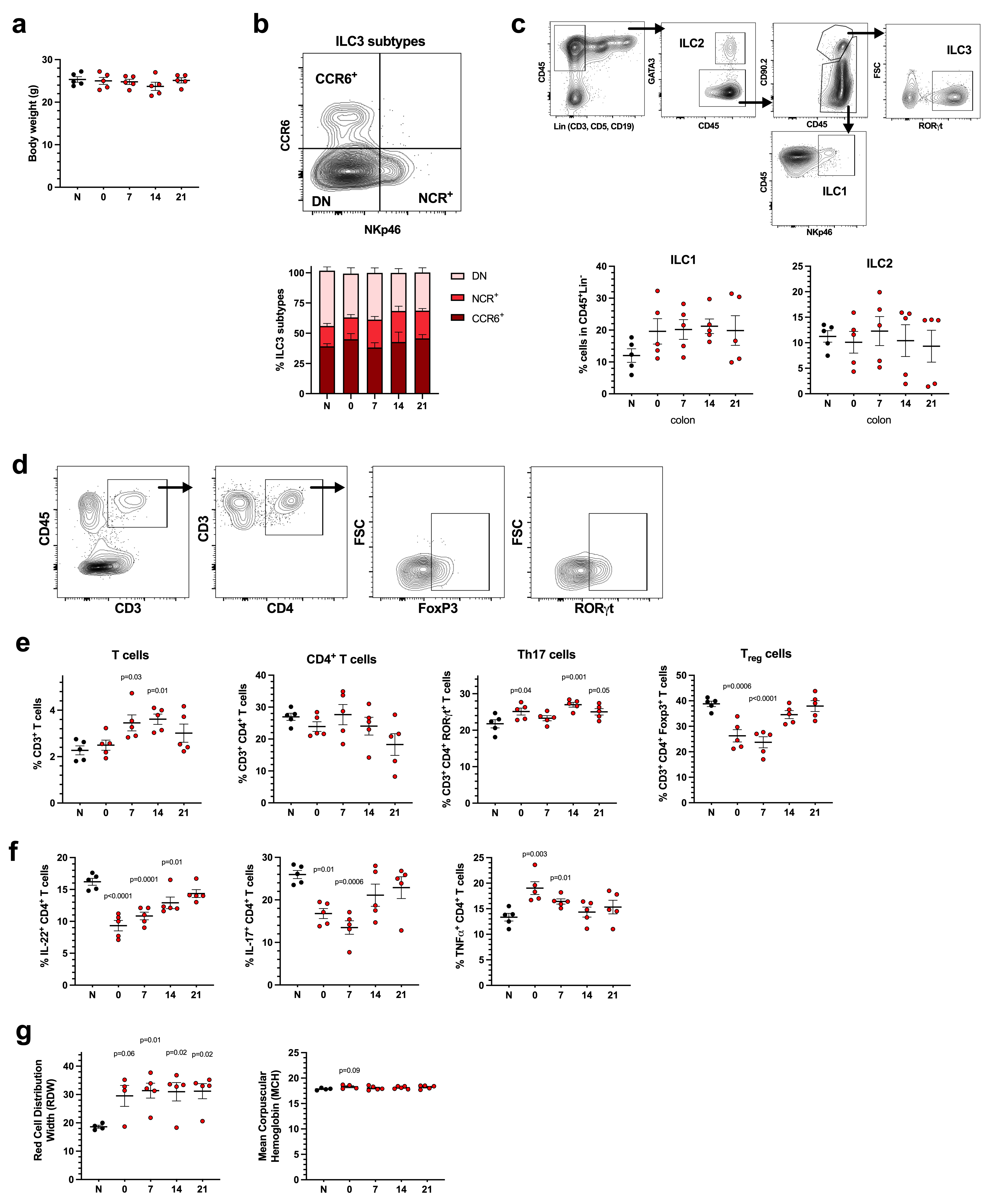


**FIG S2**

**
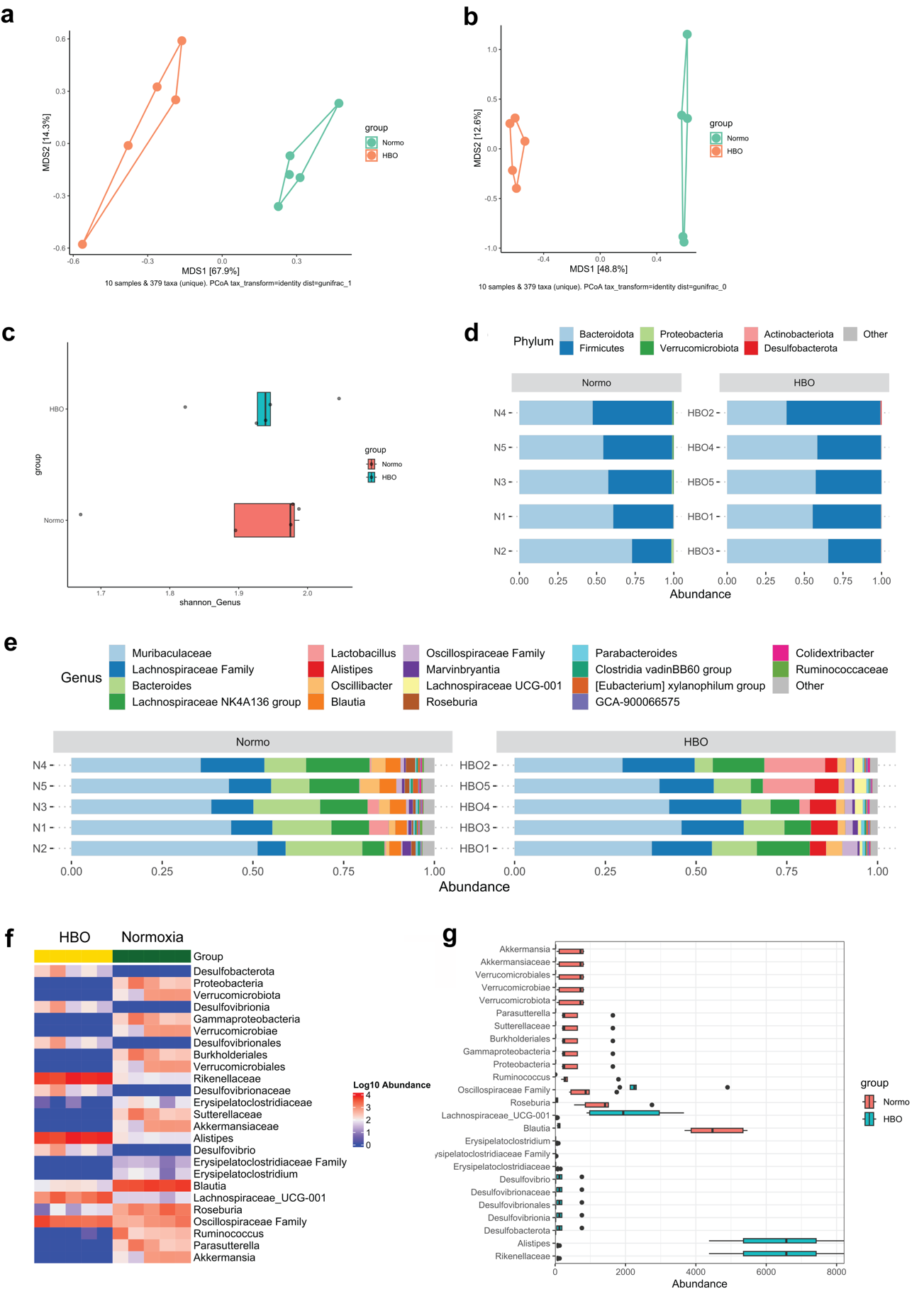
**

**FIG S3**

**FIG S4**

**
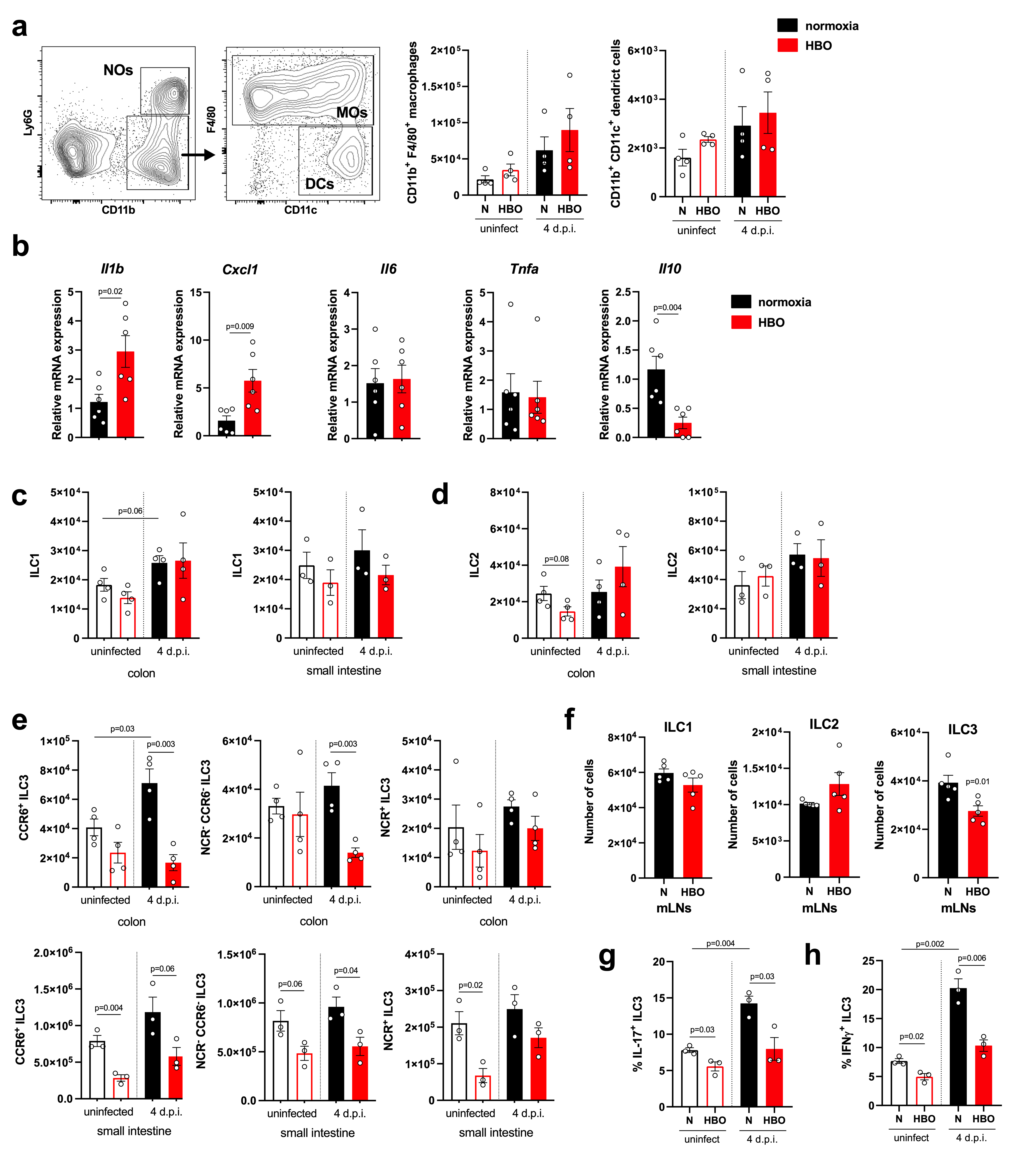
**

**FIG S5**

**
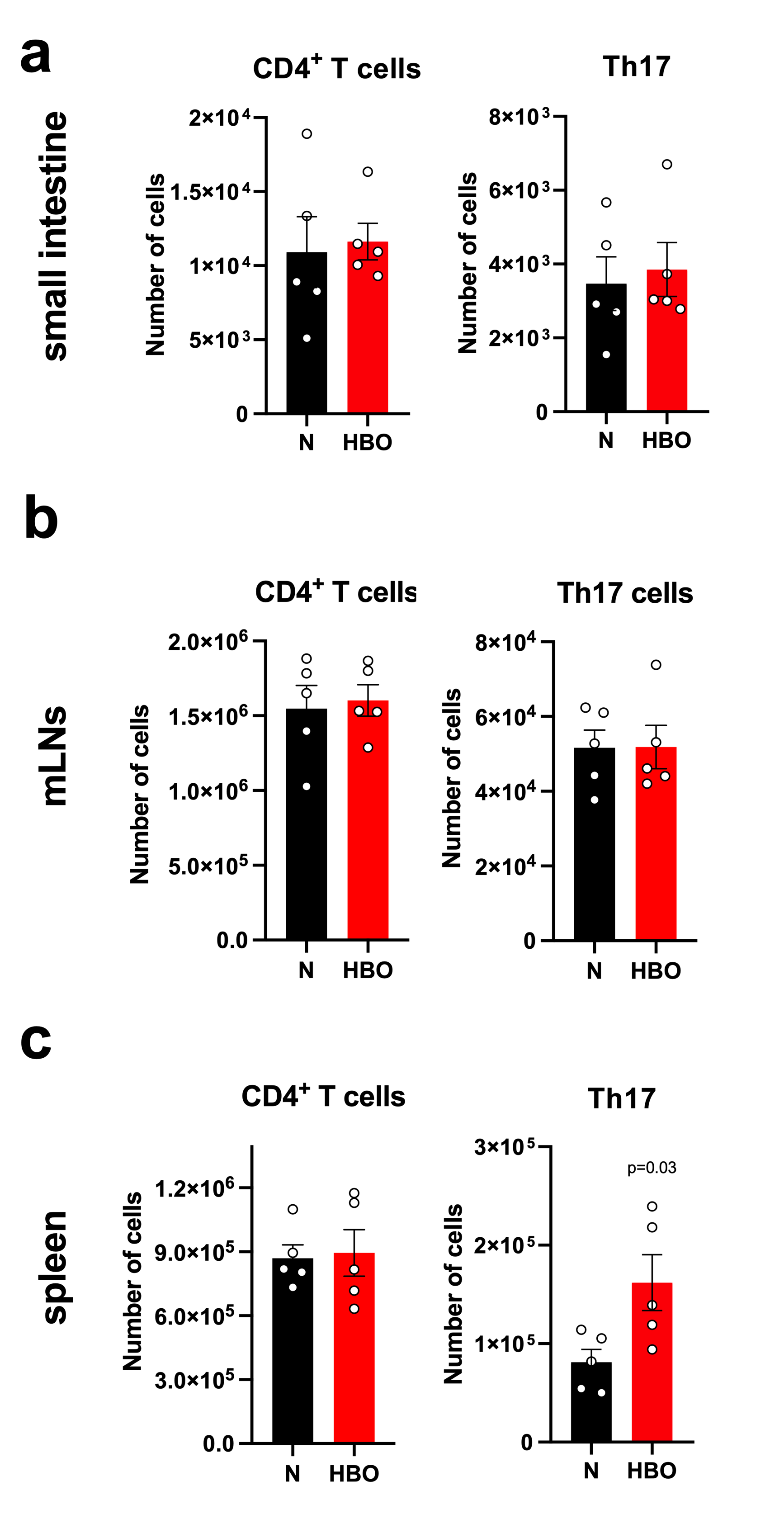
**

**FIG S6**

**
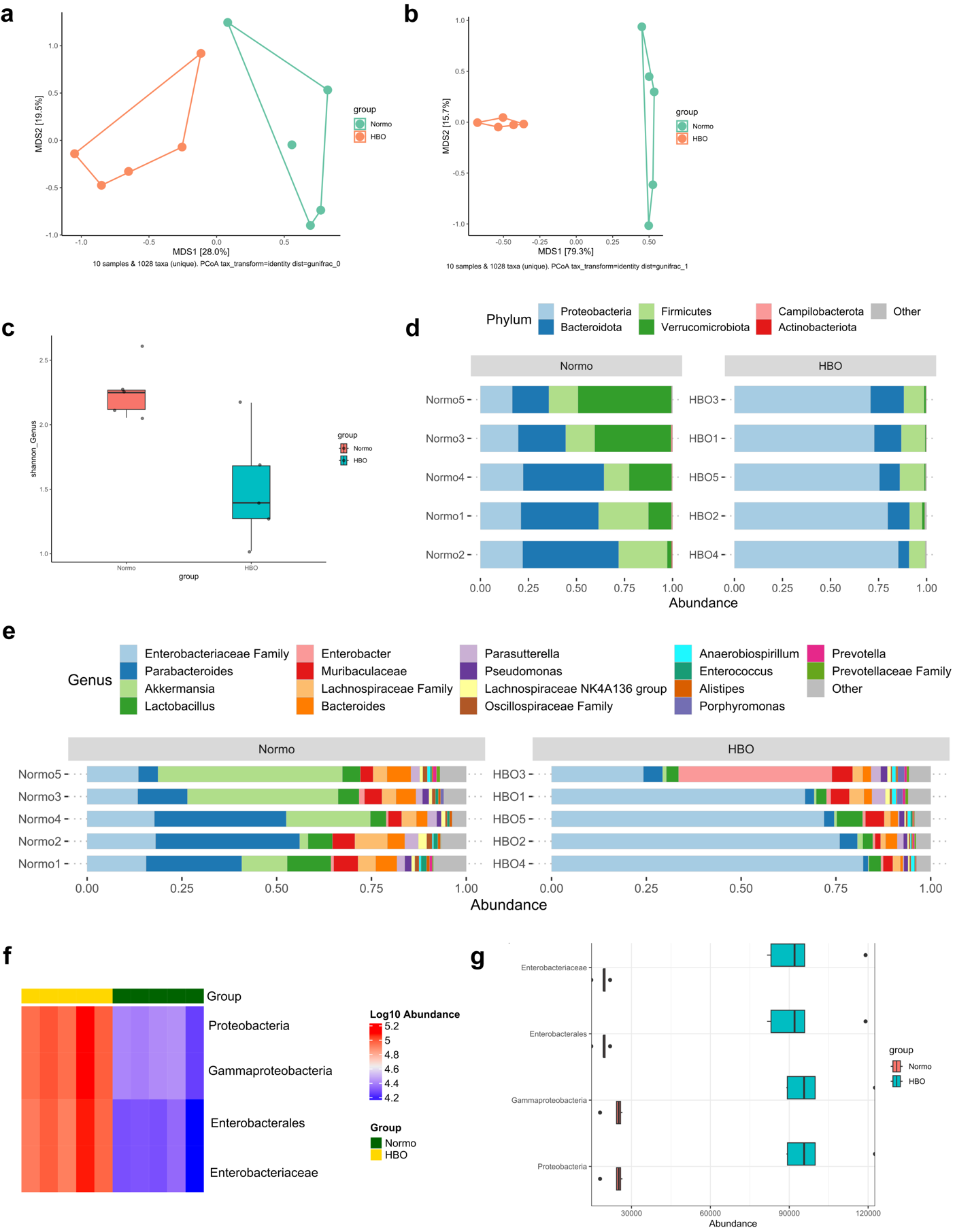
**

**FIG S7**

**
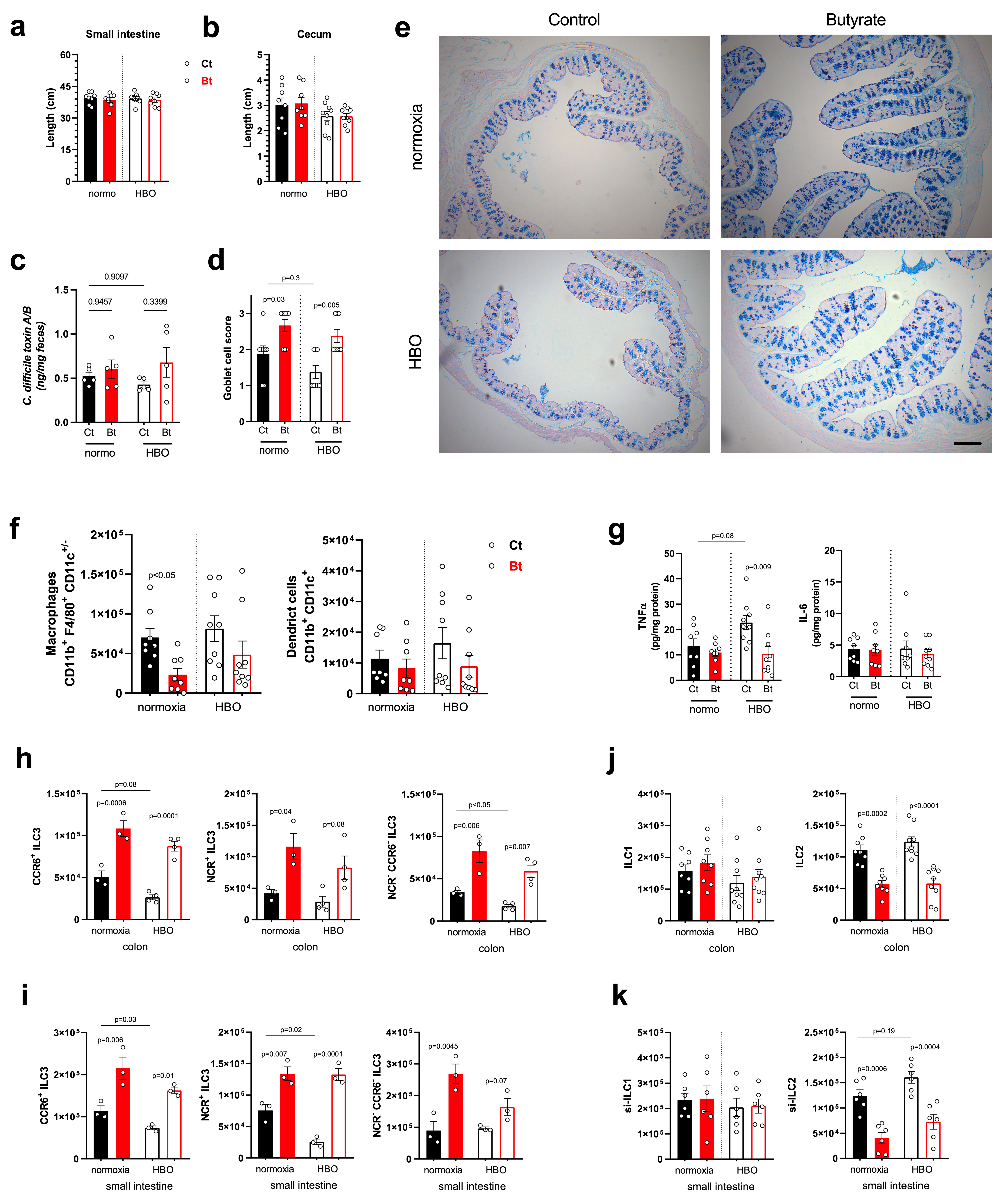
**

**FIG S8**

**
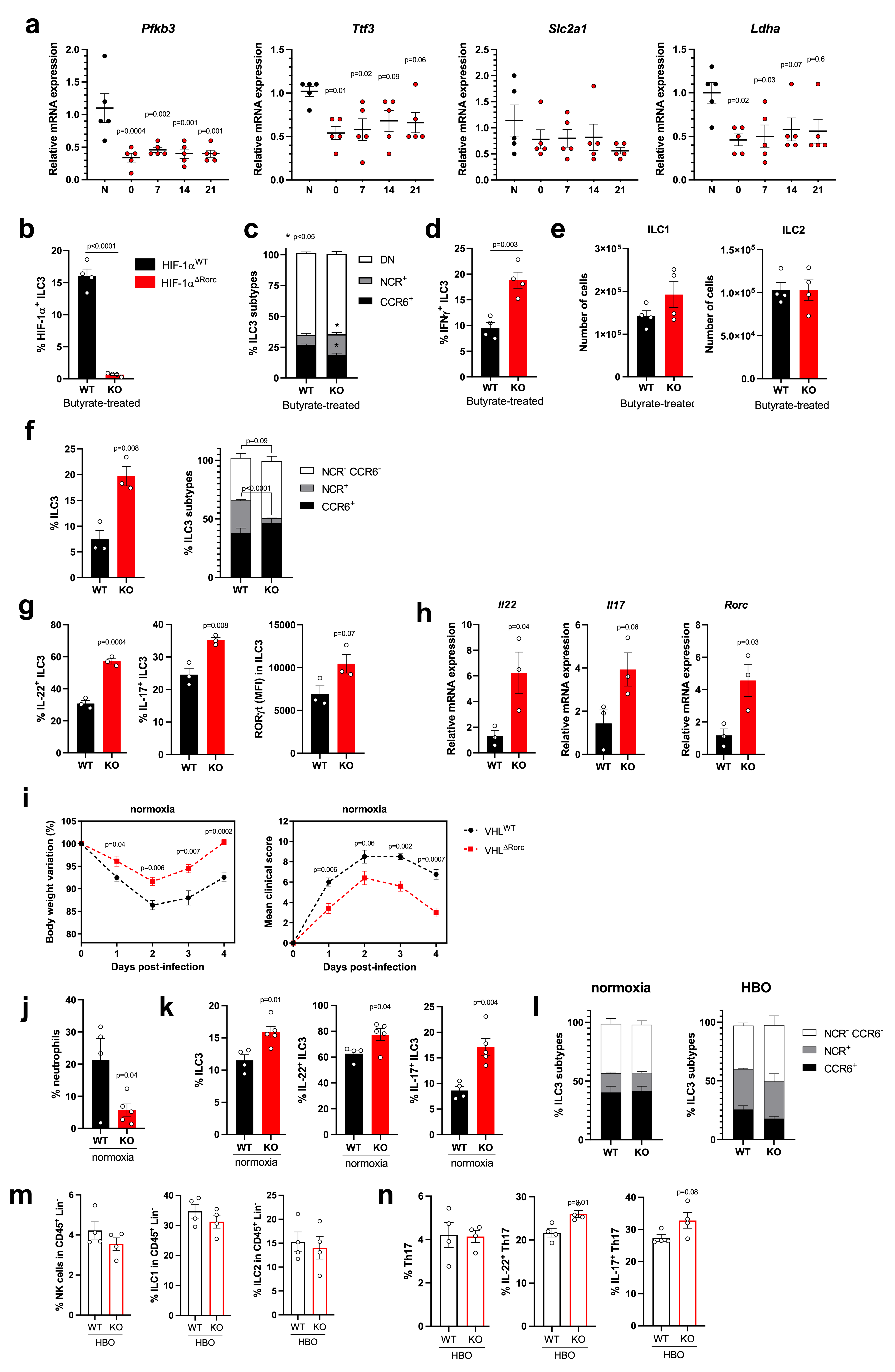
**

**FIG S9**

**
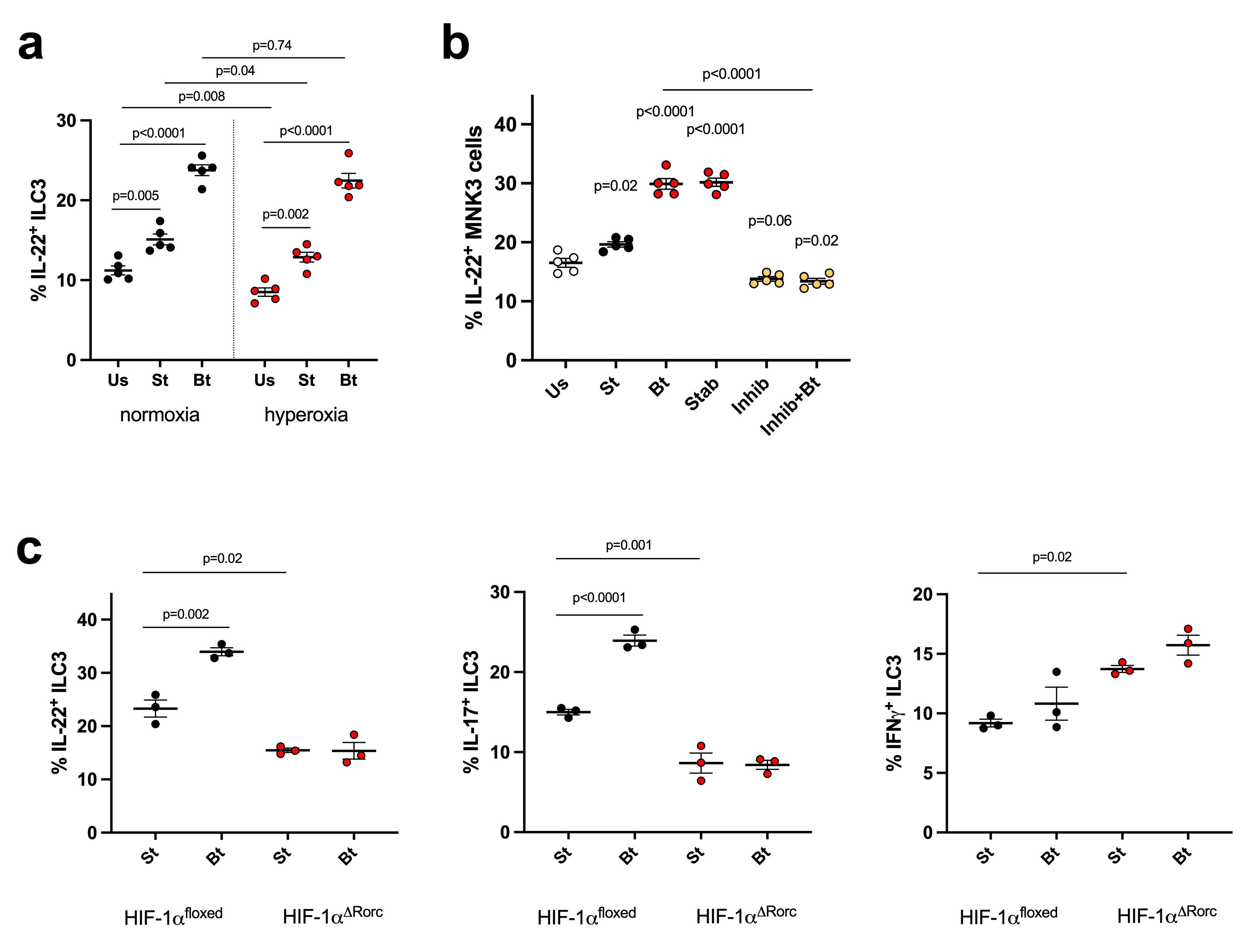
**

Supplement: Supplemental Material [file KGMI_A_2297872_SM0505.zip › SuppFigFachi1123.docx]
